# Dynamic Etching‐Induced Cl‐Terminated Ti_3_C_2_Cl_x_/Ti_3_ZnC_2_ Heterostructure for Ammonia Electrosynthesis and Zinc‐Nitrogen Batteries

**DOI:** 10.1002/advs.202524280

**Published:** 2026-02-04

**Authors:** Yu Wang, Ying Sun, Liqun Ye, Jichi Liu, Hui Li, Yang Fu, Fengzhan Sun, Jieshan Qiu, Chang Yu, Tianyi Ma

**Affiliations:** ^1^ Institute of Clean Energy Chemistry Key Laboratory for Green Synthesis and Preparative Chemistry of Advanced Materials of Liaoning Province College of Chemistry Liaoning University Shenyang Liaoning P. R. China; ^2^ College of Materials and Chemical Engineering Key Laboratory of Inorganic Nonmetallic Crystalline and Energy Conversion Materials China Three Gorges University Yichang P. R. China; ^3^ Centre For Atomaterials and Nanomanufacturing (CAN) School of Science RMIT University Melbourne Victoria Australia; ^4^ ARC Industrial Transformation Research Hub for Intelligent Energy Efficiency in Future Protected Cropping (E2Crop) Melbourne Victoria Australia; ^5^ Shanghai Advanced Research Institute Chinese Academy of Sciences Shanghai P. R. China; ^6^ College of Chemical Engineering State Key Laboratory of Chemical Resource Engineering Beijing University of Chemical Technology Beijing P. R. China; ^7^ State Key Laboratory of Fine Chemicals, Frontier Science Center for Smart Materials Liaoning Key Lab for Energy Materials and Chemical Engineering School of Chemical Engineering Dalian University of Technology Dalian Liaoning P. R. China

**Keywords:** electrocatalysis, heterostructure, nitrogen reduction reaction, Ti_3_C_2_Cl_x_ MXene, Zn‐N_2_ battery

## Abstract

2D Ti_3_C_2_T_x_ MXenes are of great potential in catalysis, energy storage, and conversion, yet the controlled tuning of their structure, intrinsic activity, and stability remains a challenge. Herein, we address this challenge through a dual‐modification strategy, synthesizing a Cl‐terminated MXene/MAX (Ti_3_C_2_Cl_x_/Ti_3_ZnC_2_) heterostructure by a dynamic etching approach for efficient electrocatalytic nitrogen reduction reaction (NRR). This catalyst achieves an NH_3_ yield of 20.1 µg h^−1^ mg^−1^ and a Faradaic efficiency of 38.1% at −0.2 V vs. RHE in 0.1 m KOH electrolyte, with high stability for over 70 h, positioning it among the top‐performing MXene‐based NRR electrocatalysts. Experimental and theoretical analyses demonstrate that the Ti_3_C_2_Cl_x_/Ti_3_ZnC_2_ heterostructure modulates the electronic structure of Ti sites, thus optimizing the intermediate adsorption and reducing the energy barrier of *NH_2_ → NH_3_ conversion in the distal pathway to 0.7 eV. The Zn‐N_2_ battery assembled with Ti_3_C_2_Cl_x_/Ti_3_ZnC_2_ can reach a peak power density of 36.5 µW cm^−2^, with an NH_3_ yield of 13.1 µg h^−1^ mg^−1^. This study has demonstrated that the dual modification strategy involving surface terminations regulation and heterostructure construction is effective to improve both NRR activity and stability of MXene‐based electrocatalysts, which is crucial for efficient NH_3_ production and energy generation. This improvement paves the way for efficient ammonia and energy co‐generation, providing a viable materials design strategy and deeper mechanistic insights.

## Graphical abstract

A stable Ti_3_C_2_Cl_x_/Ti_3_ZnC_2_ heterostructure, fabricated by dynamic etching, exhibits outstanding electrocatalytic NRR activity (NH_3_ yield: 20.1 µg h^−1^ mg^−1^, FE: 38.1%) in alkaline media. The corresponding Zn‐N_2_ battery achieves a peak power density of 36.5 µW cm^−2^ and an NH_3_ yield of 13.1 µg h^−1^ mg^−1^, providing an advanced strategy for NH_3_ and electricity cogeneration.

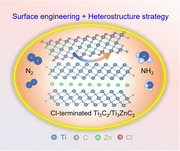

## 1 Introduction

Ammonia (NH_3_) is a vital industrial raw material and a promising carbon‐free energy storage intermediate, with a global annual market exceeding 170 million tons [1, 2, 3]. At present, ca. 90% of NH_3_ is produced by the high‐energy Haber–Bosch process, which requires severe operating conditions (400–500°C, 150–350 atm), resulting in substantial fossil fuel consumption and significant CO_2_ emissions [4, 5, 6]. By contrast, N_2_ electroreduction reaction (NRR) in aqueous media enables sustainable NH_3_ production under ambient conditions, powered by renewable energy with H_2_O as the hydrogen donor [7]. Unfortunately, the combined challenges of N≡N bond activation (941 kJ mol^−1^) and hydrogen evolution reaction (HER) competition severely limit achievable NH_3_ yield and Faradaic efficiency (FE) in NRR systems, impeding the large‐scale applications of the electrocatalytic NRR [8, 9, 10]. As such, there is an urgent need to design high‐efficiency NRR electrocatalysts for NH_3_ synthesis.

MXenes have shown great potential in electrocatalytic NRR owing to the compact multilayered structure, outstanding electrical conductivity, and tunable surface terminations [11, 12, 13]. It has been found that the edge‐exposed Ti atoms in Ti_3_C_2_T_x_ serve as active sites for nitrogen activation [14]. However, conventional synthesis of MXenes relies on hazardous HF etching, which typically results in surface terminations dominated by ─O, ─OH, and particularly inert ─F groups, thus leading to poor electrochemical NRR performance (FE < 10%) [15, 16]. Previously, we have synthesized Fe loaded on fluorine‐free Ti_3_C_2_T_x_ that can deliver an NH_3_ yield of 18.25 µg h^−1^ mg^−1^ yet with an unsatisfactory stability of only 24 h [17]. Therefore, simultaneously enhancing both the stability and activity of Ti_3_C_2_T_x_‐based electrocatalysts remains a significant challenge.

Heterostructure engineering is effective to boost the intrinsic activity of MXene‐based catalysts by enhancing active site accessibility, modulating the electronic state, and synergistically regulating the adsorption energetics of key intermediates [18, 19]. For instance, a Ti_3_C_2_ MXene/Ti_3_AlC_2_ MAX heterostructure was fabricated by precisely adjusting the LiF percentage in a mixed etching solution (LiF + HCl), which showed an NH_3_ yield of 2.73 µg h^−1^ cm^−2^ with a high FE of 36.9% [20], showcasing the advantages of heterostructure construction for developing highly active and selective MXene‐based electrocatalysts. Moreover, previous studies showed that the change of A‐site atoms can alter the bonding strength and electronic structure, further improving the stability of the MAX phase [21, 22]. Therefore, constructing a heterostructure by coupling MAX phase and Cl‐terminated MXene could provide unprecedented improvements in both structural stability and catalytic performance for Ti_3_C_2_T_x_ electrocatalysts, which have not been reported before.

Herein, we report a dynamic etching approach to fabricate a Cl‐terminated MXene/Zn‐MAX (Ti_3_C_2_Cl_x_/Ti_3_ZnC_2_) heterostructure for efficient and continuous NRR. The optimized Ti_3_C_2_Cl_x_/Ti_3_ZnC_2_ catalyst exhibits a favorable NH_3_ yield of 20.1 µg h^−1^ mg^−1^ and a high FE of 38.1% at −0.2 V vs. RHE in 0.1 m KOH with robust stability exceeding 70 h, surpassing most reported MXene‐based NRR electrocatalysts. Spectroscopic analysis and theoretical calculations demonstrate that the construction of the Ti_3_C_2_Cl_x_/Ti_3_ZnC_2_ heterostructure effectively regulates the charge distribution on the heterointerfaces and optimizes the adsorption capacity of Ti for each intermediate for facilitating NH_3_ formation. Impressively, the Ti_3_C_2_Cl_x_/Ti_3_ZnC_2_ enables a Zn‐N_2_ battery that delivers a peak power density of 36.5 µW cm^−2^ and an NH_3_ yield of 13.1 µg h^−1^ mg^−1^. This work not only outlines a dual‐modification strategy that employs heterointerface engineering and termination regulation to develop efficient MXene‐based electrocatalysts, but also provides a “one‐stone‐two‐birds” strategy for simultaneous NH_3_ and electrical energy production.

## 2 Results and Discussion

### 2.1 Characterizations

**FIGURE 1:**
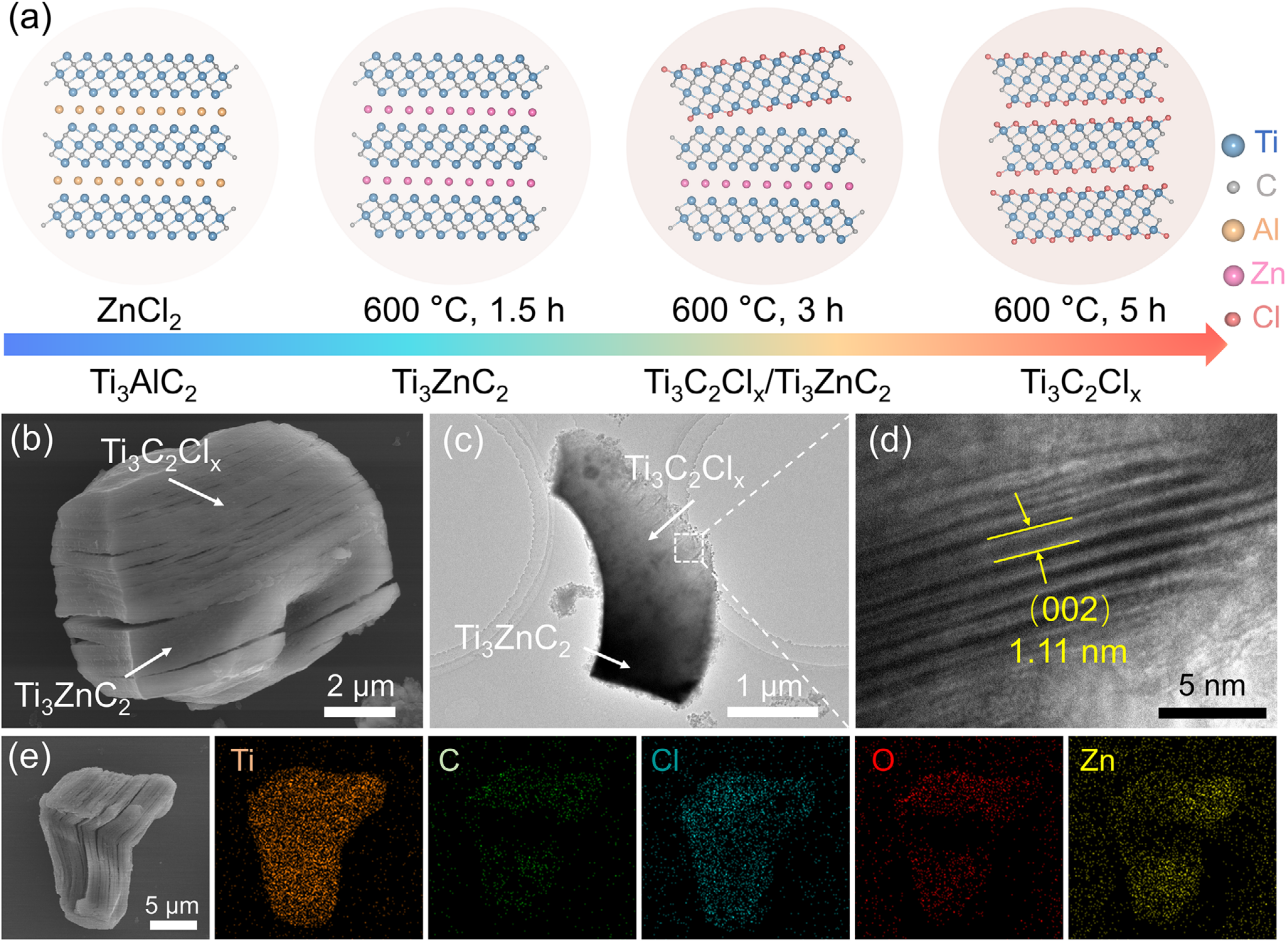
(a) Schematic of the synthesis of Ti_3_ZnC_2_, Ti_3_C_2_Cl_x_/Ti_3_ZnC_2_ heterostructure, and Ti_3_C_2_Cl_x_ at different etching times. (b) SEM, (c) TEM, (d) HRTEM, (e) EDS elemental mapping images of Ti_3_C_2_Cl_x_/Ti_3_ZnC_2_ heterostructure.

The Cl‐terminated heterostructures were synthesized via a dynamic etching approach. Unlike conventional HF etching, this strategy facilitates a time‐controlled, progressive phase transformation, wherein ZnCl_2_ molten salt gradually replaces Al layers and introduces Cl terminations, driving the sequential structural evolution: Ti_3_AlC_2_ → Ti_3_ZnC_2_ (1.5 h) → Ti_3_C_2_Cl_x_/Ti_3_ZnC_2_ heterostructure (3 h) → Ti_3_C_2_Cl_x_ (5 h). Figure 1a schematically illustrates the time‐dependent structural evolution during the dynamic etching process. Treating Ti_3_AlC_2_ with ZnCl_2_ at a molar ratio of 1:6 at 600°C, enabled the precise synthesis of distinct phases by controlling the etching duration: Ti_3_ZnC_2_ at 1.5 h, Ti_3_C_2_Cl_x_/Ti_3_ZnC_2_ heterostructure at 3 h, and fully etched Ti_3_C_2_Cl_x_ MXene at 5 h. This controlled phase transformation underscores the efficacy of our etching strategy. The scanning electron microscopy (SEM) was employed to examine morphological changes. The original Ti_3_AlC_2_ particles have a typical densely layered structure (Figure S1). After 1.5 h of etching, the Ti_3_ZnC_2_ (Figure S2) retains a layered structure similar to that of Ti_3_AlC_2_. This implies that the Al‐to‐Zn substitution reaction occurs at the atomic level in the molten salt medium, generating minimal damage to the hexagonal crystal structure of the MAX phase [21]. The energy‐dispersive X‐ray spectroscopy (EDS) analysis of Ti_3_ZnC_2_ confirms a Ti:Zn:C atomic ratio of 18.49:15.63:51.27, with a trace amount of residual Al (0.8 at%), evidencing the near‐complete replacement of Al by Zn. The pivotal Ti_3_C_2_Cl_x_/Ti_3_ZnC_2_ heterostructure, obtained at 3 h, displays a distinctive “semi‐open” layered architecture (Figure 1b; Figure S3), suggesting the partial etching of the Ti_3_ZnC_2_ phase. With prolonged etching to 5 h, the MAX phase is entirely etched to form Ti_3_C_2_Cl_x_ MXene with an obvious accordion‐like morphology and expanded interlayer spacing (Figure S4a). The EDS analysis of Ti_3_C_2_Cl_x_ confirms the substantial removal of Zn and Al and the successful introduction of Cl terminations, as evidenced by its elemental composition: Ti:C:Cl:O = 39.49:20.15:18.91:19.49 (atomic ratio), alongside only trace Zn (0.11 at%) and Al (1.84 at%) (Figure S4b,c).

The microstructure was further elucidated by transmission electron microscopy (TEM) and high‐resolution TEM (HRTEM). The TEM image (Figure 1c) visually confirms the “semi‐open” structure of the Ti_3_C_2_Cl_x_/Ti_3_ZnC_2_ heterostructure. The HRTEM image (Figure 1d) reveals a well‐defined lattice fringe with an interlayer spacing of 1.11 nm, corresponding to the (002) plane of Ti_3_C_2_ [23]. The intimate interface between Ti_3_C_2_Cl_x_ and Ti_3_ZnC_2_ suggests the potential for interfacial charge redistribution, a key factor in enhancing electrocatalytic activity. Additionally, the EDS elemental mapping analysis (Figure 1e) confirms the homogeneous distribution of Cl terminations on the Ti_3_C_2_Cl_x_/Ti_3_ZnC_2_ heterostructure surface.

**FIGURE 2:**
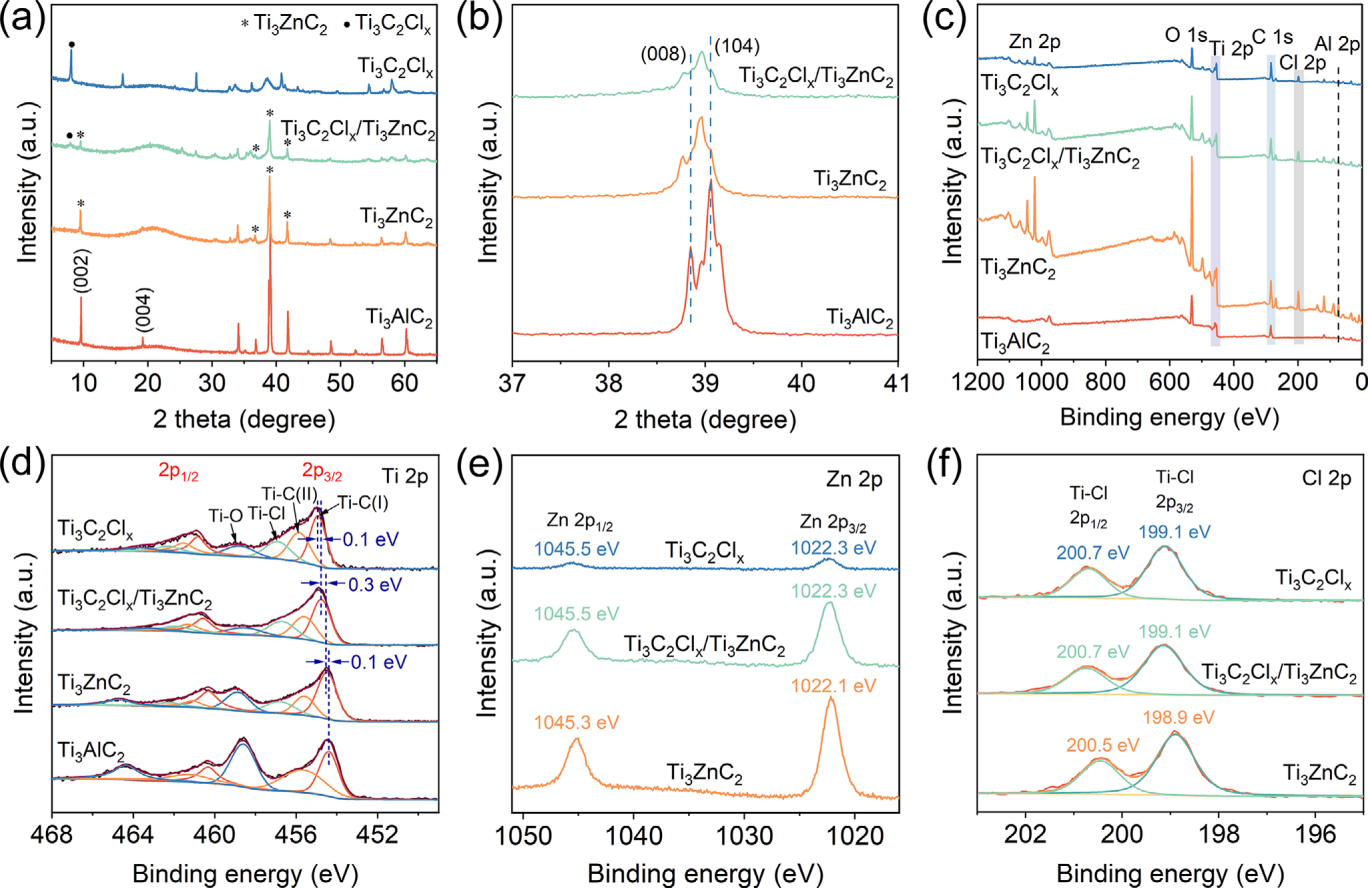
(a, b) XRD patterns, XPS spectra of Ti_3_AlC_2_, Ti_3_ZnC_2_, Ti_3_C_2_Cl_x_/Ti_3_ZnC_2_ heterostructure, and Ti_3_C_2_Cl_x_ in (c) survey scan, (d) Ti 2p, (e) Zn 2p, and (f) Cl 2p region, respectively.

The crystalline phase evolution was analyzed by X‐ray diffraction (XRD). The main diffraction peaks at 9.6, 19.2, 38.8, and 39.1° correspond to the (002), (004), (008), and (104) planes of Ti_3_AlC_2_ MAX phase, respectively (Figure 2a). At the etching time of 1.5 h (Figure 2b, orange curve), the (008) and (104) peaks shift to lower angles compared to those of Ti_3_AlC_2_ due to the replacement of Al by Zn atoms, leading to an increased lattice constant, indicating the formation of Ti_3_ZnC_2_ [24]. At the etching time of 3 h (Figure 2a, green curve), the (002) peak shifts toward a lower angle, suggesting the successful formation of Ti_3_C_2_Cl_x_ MXene [24]. The existence of diffraction peaks from both Ti_3_ZnC_2_ and Ti_3_C_2_Cl_x_ at this stage provides definitive evidence for the successful construction of the Ti_3_C_2_Cl_x_/Ti_3_ZnC_2_ heterostructure. After prolonging the etching time to 5 h (Figure 2a, blue curve), the (104) characteristic peak of Ti_3_ZnC_2_ nearly disappears, and the intensity of Ti_3_C_2_Cl_x_ increases significantly, confirming the complete transformation of Ti_3_ZnC_2_ to Ti_3_C_2_Cl_x_. The increased interlayer spacing enables more active sites to be exposed, which is favorable for nitrogen electroreduction.

X‐ray photoelectron spectroscopy (XPS) survey scans (Figure 2c) confirm the presence of Ti, C, and O in all samples. As the etching time increases, the Zn content varies in a distinct volcano‐shaped trend, perfectly mirroring the sequential phase transformation: Ti_3_AlC_2_ → Ti_3_ZnC_2_ → Ti_3_C_2_Cl_x_/Ti_3_ZnC_2_ heterostructure → Ti_3_C_2_Cl_x_. High‐resolution spectra offer deep insights into the chemical states and electronic interactions. In the Ti 2p spectra (Figure 2d), the Ti─C(I) (2p_1/2_ at ∼460 eV and 2p_3/2_ at ∼455 eV) and Ti─C(II) (2p_1/2_ at ∼461 eV and 2p_3/2_ at ∼456 eV) bonds are associated with the TiC_6_ octahedral building blocks [25, 26]. The formation of Ti─Cl (2p_1/2_ at ∼462 eV and 2p_3/2_ at ∼457 eV) and Ti─O (2p_1/2_ at ∼463 eV and 2p_3/2_ at ∼458 eV) bonds can be ascribed to the Cl and O terminals, where the Cl atoms originate from the molten salt etching process, while the O atoms may arise from the washing process and surface oxidation [17, 27]. Interestingly, compared to pristine Ti_3_AlC_2_, the Ti_3_ZnC_2_ exhibits a positive binding energy shift for the Ti─C(I) 2p_3/2_ (∼0.1 eV) owing to the substitution of Al atoms by Zn and the incorporation of surface Cl‐terminals. Remarkably, the formation of the Ti_3_C_2_Cl_x_/Ti_3_ZnC_2_ heterostructure induces a ∼0.3 eV positive shift in the Ti─C(I) 2p_3/2_ peak position relative to pristine Ti_3_ZnC_2_, suggesting the interfacial electron transfer between Ti_3_ZnC_2_ and Ti_3_C_2_Cl_x_. Furthermore, the Ti─C(I) 2p_3/2_ of the obtained Ti_3_C_2_Cl_x_ shows a positive shift (∼0.1 eV) compared to Ti_3_C_2_Cl_x_/Ti_3_ZnC_2_ due to the extraction of Zn atoms from Ti_3_ZnC_2_ [28]. This significant binding energy shift serves as direct evidence of interfacial electron transfer, which is essential for optimizing the adsorption energetics of NRR intermediates through electronic modulation. Concurrently, the decreased intensity of the Ti─O peak in the heterostructure suggests enhanced oxidation resistance and structural stability [28]. The Zn 2p spectra of all samples (Figure 2e) exhibit characteristic peaks corresponding to Zn 2p_3/2_ and Zn 2p_1/2_, originating from the oxide layer on Zn [24]. In the Cl 2p spectra (Figure 2f), the binding energies of 198.5 and 200.0 eV correspond to Ti─Cl 2p_3/2_ and Ti─Cl 2p_1/2_ [29, 30], respectively, further confirming the formation of Cl terminals. All above characterization analyses corroborate the successful and controllable preparation of Ti_3_C_2_Cl_x_/Ti_3_ZnC_2_ heterostructure via a dynamic etching approach.

### 2.2 Nitrogen Electroreduction

**FIGURE 3:**
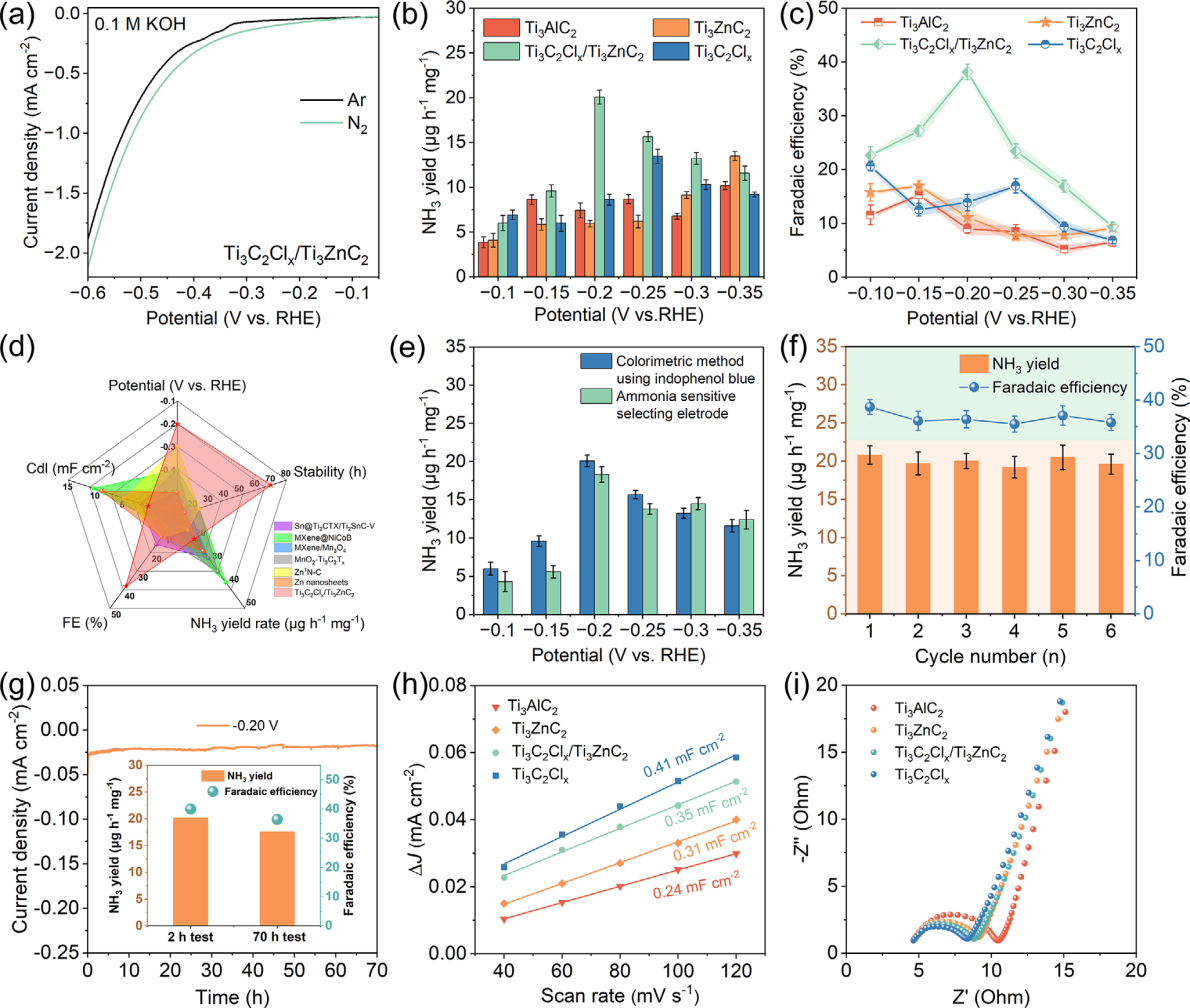
(a) LSV curves of Ti_3_C_2_Cl_x_/Ti_3_ZnC_2_ heterostructure in Ar‐ and N_2_‐saturated 0.1 m KOH. (b) NH_3_ yields and (c) FEs at various potentials. (d) Comparison of NRR performance with several reported electrocatalysts. (e) Comparison of NH_3_ yields determined by different methods. (f) NH_3_ yields and FEs in six consecutive cycles. (g) Chronoamperometry results (inset: FEs and NH_3_ yields in 2 and 70 h). (h) C_dl_ measurement of all samples. (i) Nyquist plots of all samples.

The N_2_ electroreduction performance of the Ti_3_C_2_Cl_x_/Ti_3_ZnC_2_ heterostructure was investigated in a meticulously purged H‐cell. Initially, linear sweep voltammetry (LSV) tests for the Ti_3_C_2_Cl_x_/Ti_3_ZnC_2_ heterostructure were performed in 0.1 m KOH with continuous purging of either N_2_ or Ar to access the electrocatalytic NRR activity (Figure 3a). It can be observed that the LSV curve in N_2_‐saturated electrolyte displays a significantly higher current density than that in Ar‐saturated electrolyte when the potentials are below −0.1 V vs. RHE. The significant difference clearly confirms the prominent NRR activity of the Ti_3_C_2_Cl_x_/Ti_3_ZnC_2_ heterostructure, beyond the contribution from the competing HER. To evaluate the N_2_ electroreduction activity, chronoamperometric tests were performed for 2 h at various potentials. The produced NH_3_ was quantified by the indophenol blue method [31], and the by‐product hydrazine (N_2_H_4_) was detected via Watt–Chrisp method [32]. Notably, N_2_H_4_ was undetectable after 2 h of electrolysis in N_2_‐saturated electrolyte (Figure S5) based on the standard calibration curve of N_2_H_4_ (Figure S6), demonstrating an exceptional selectivity of the Ti_3_C_2_Cl_x_/Ti_3_ZnC_2_ heterostructure toward the desirable NH_3_ product, a critical metric for practical NRR application. The standard curve of NH_3_ measured by the indophenol blue method is shown in FigureS7.

A comprehensive comparison of the NRR catalytic capacity is presented in Figure 3b,c. Among all catalysts, the Ti_3_C_2_Cl_x_/Ti_3_ZnC_2_ heterostructure delivers the highest NH_3_ yield of 20.1 µg h^−1^ mg^−1^ with a high FE of 38.1% at a low overpotential of −0.2 V vs. RHE. This performance significantly surpasses that of Ti_3_AlC_2_ (10.2 µg h^−1^ mg^−1^, 15.2%), Ti_3_ZnC_2_ (13.5 µg h^−1^ mg^−1^, 17.0%), and Ti_3_C_2_Cl_x_ (14.5 µg h^−1^ mg^−1^, 20.6%) under the same conditions (Figures S8–S11). The results show that the N_2_ electroreduction activity can be enhanced by substituting the Al atomic layers with the Zn atomic layers in the MAX phase. In comparison to the MAX phase, the fully etched Ti_3_C_2_Cl_x_ MXene exhibits superior NRR electrocatalytic activity owing to more exposed Ti active sites. Furthermore, the Ti_3_C_2_Cl_x_/Ti_3_ZnC_2_ heterostructure with Cl terminals on the surface exhibits significantly enhanced electrocatalytic NRR activity compared to the corresponding MAX and MXene. This enhancement is primarily attributed to the unique heterostructure and surface Cl terminals, which facilitate the conversion of reaction intermediates by tuning the electronic structure of Ti active sites, as evidenced by the Ti 2p XPS spectrum and subsequent density functional theory (DFT) calculations. As expected, a notable decrease in FE at potentials below −0.2 V vs. RHE is ascribed to the increased competitive HER at higher overpotentials (Figure 3c) [33]. To our knowledge, the FE, stability, and overpotential of the Ti_3_C_2_Cl_x_/Ti_3_ZnC_2_ heterostructure are significantly better than most reported MXene‐based catalysts and other state‐of‐the‐art NRR electrocatalysts (Figure 3d; Table S1) [34, 35, 36, 37, 38, 39, 40, 41, 42].

To validate the accuracy of the colorimetric methods, NH_3_ concentrations were also measured using the ammonia gas‐sensitive electrode method [43]. The results shown in Figure 3e and Figure S12 largely corroborate those stemming from the indophenol blue method, affirming the reliability of the catalyst performance assessment. Afterward, a series of control experiments was done to confirm that NH_3_ was produced by N_2_ electroreduction on the Ti_3_C_2_Cl_x_/Ti_3_ZnC_2_ heterostructure. As depicted in Figure S13, a high concentration of NH_3_ is detected only in N_2_‐saturated electrolyte at −0.2 V vs. RHE, verifying that the detected ammonia is obtained through nitrogen electroreduction over Ti_3_C_2_Cl_x_/Ti_3_ZnC_2_ heterostructure, rather than by contamination from the catalyst itself, electrolyte, experimental environment, reaction apparatus, or the membrane. Furthermore, the NRR performance of the Ti_3_C_2_Cl_x_/Ti_3_ZnC_2_ electrocatalyst was also examined in acidic (0.1 m HCl) and neutral (0.1 m Na_2_SO_4_) electrolytes (Figure S14). In 0.1 m Na_2_SO_4_ electrolyte, the Ti_3_C_2_Cl_x_/Ti_3_ZnC_2_ heterostructure achieves a FE of 4.5% and a peak NH_3_ yield of 4.4 µg h^−1^ mg^−1^ at −0.4 V vs. RHE (Figures S15 and S16). In 0.1 m HCl electrolyte, it achieved an optimal NH_3_ yield of 13.6 µg h^−1^ mg^−1^ at −0.1 V vs. RHE and a maximum FE of 4.6% at −0.05 V vs. RHE (Figures S17 and S18). The NH_3_ yield and FE of Ti_3_C_2_Cl_x_/Ti_3_ZnC_2_ heterostructure in 0.1 m KOH are higher than those in 0.1 m HCl and 0.1 m Na_2_SO_4_ electrolytes. This enhancement is attributed to the limited proton availability and an altered HER equilibrium in the alkaline media that suppresses the competing HER side reaction [44, 45].

The operational stability of the Ti_3_C_2_Cl_x_/Ti_3_ZnC_2_ heterostructure, a critical parameter for practical application, was examined through successive cycling and long‐term chronoamperometric tests. As shown in Figure 3f and Figure S19, both the NH_3_ yields and FEs are consistently maintained during six successive electrolytic cycles at −0.2 V vs. RHE. Specifically, NH_3_ yields remain consistent at 19 µg h^−1^ mg^−1^ with FEs over 35% throughout the cycles, suggesting the high stability of the Ti_3_C_2_Cl_x_/Ti_3_ZnC_2_ heterostructure. More strikingly, the Ti_3_C_2_Cl_x_/Ti_3_ZnC_2_ exhibited excellent stability over a 70‐h chronoamperometric test, with minimal loss in the NH_3_ production rate and Faradaic efficiency, and a steady current density. (Figure 3g). Following the durability assessment, the original “semi‐open” heterostructure morphology and crystalline integrity were preserved, confirmed by SEM (Figure S20), TEM, and HRTEM results (Figure S21), highlighting its remarkable structural stability.

To further elucidate the NRR activity of the Ti_3_C_2_Cl_x_/Ti_3_ZnC_2_ heterostructure, the electrochemical double‐layer capacitance (C_dl_) was measured to evaluate the electrochemically active surface area (ECSA) of each sample (Figure 3h; Figure S22). Compared to Ti_3_AlC_2_ (0.24 mF cm^−2^), Ti_3_ZnC_2_ (0.31 mF cm^−2^) has a slightly higher ECSA, indicating that Ti_3_ZnC_2_ has more exposed active sites, which contribute to the enhancement of NRR activity [46, 47]. Ti_3_C_2_Cl_x_ (0.41 mF cm^−2^) shows a higher ECSA than that of Ti_3_C_2_Cl_x_/Ti_3_ZnC_2_ heterostructure (0.35 mF cm^−2^), suggesting that the fully etched Ti_3_C_2_Cl_x_ has more exposed Ti active sites. However, the Ti active sites may also promote the competing HER, which might diminish the NRR activity of Ti_3_C_2_Cl_x_ to some degree [20]. Additionally, the charge transfer resistance of Ti_3_C_2_Cl_x_/Ti_3_ZnC_2_ lies between that of Ti_3_C_2_Cl_x_ and Ti_3_ZnC_2_, with Ti_3_ZnC_2_ showing the highest value, suggesting that the Ti_3_C_2_Cl_x_ facilitates the electron transfer (Figure 3i). To sum up, the outstanding NRR performance of the Ti_3_C_2_Cl_x_/Ti_3_ZnC_2_ heterostructure can be ascribed to its unique heterostructure and Cl terminals, which endow the electrocatalyst with adequate NRR active sites. Meanwhile, Ti_3_C_2_Cl_x_ has good conductivity, which facilitates rapid electron transfer, thus enhancing the NRR activity of the Ti_3_C_2_Cl_x_/Ti_3_ZnC_2_ heterostructure.

### 2.3 Theoretical Calculations

**FIGURE 4:**
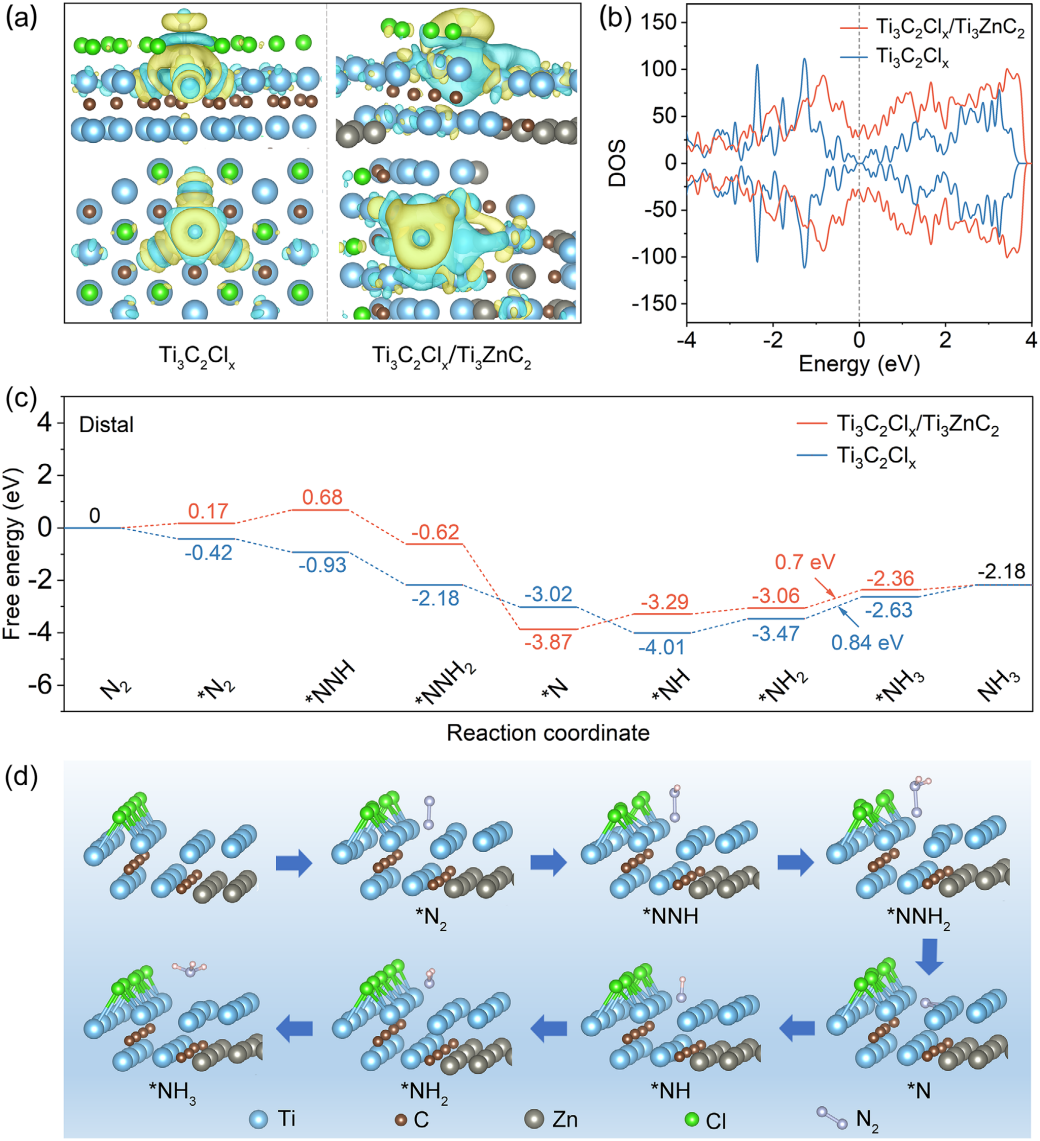
(a) Charge density differences of Ti_3_C_2_Cl_x_ and Ti_3_C_2_Cl_x_/Ti_3_ZnC_2_ heterostructure with N_2_ adsorbate in side and top view. (b) DOS of Ti_3_C_2_Cl_x_ and Ti_3_C_2_Cl_x_/Ti_3_ZnC_2_ heterostructure. (c) Calculated free energy diagram via the distal pathway on Ti_3_C_2_Cl_x_/Ti_3_ZnC_2_ heterostructure and Ti_3_C_2_Cl_x_. (d) The adsorption configurations of the key intermediates during NRR via the distal pathway.

The enhanced N_2_ electroreduction activity of Ti_3_C_2_Cl_x_/Ti_3_ZnC_2_ heterostructure was elucidated by DFT calculations. Given the inferior NRR activity of Ti_3_AlC_2_ and Ti_3_ZnC_2_ MAX phases, we focused our computational efforts on comparing the catalytic properties of the Ti_3_C_2_Cl_x_/Ti_3_ZnC_2_ heterostructure with Ti_3_C_2_Cl_x_. The theoretical models of Ti_3_C_2_Cl_x_/Ti_3_ZnC_2_ heterostructure and Ti_3_C_2_Cl_x_ were then constructed with the experimental results as reference (Figure S23). We first calculated the initial N_2_ adsorption and activation, a critical step in NRR. The charge density difference plot for N_2_ adsorption (Figure 4a) reveals pronounced charge accumulation (yellow) and depletion (cyan) regions between the N_2_ molecule and the interfacial Ti sites of the Ti_3_C_2_Cl_x_/Ti_3_ZnC_2_ heterostructure, signifying strong electronic coupling. The significant interaction is quantitatively confirmed by Bader charge analysis, which shows that the N_2_ molecule gains a substantially larger number of electrons (1.46 e^−^) when adsorbed on the heterostructure compared to on Ti_3_C_2_Cl_x_ (0.6 e^−^). This massive electron transfer from the heterostructure effectively populates the N_2_ antibonding orbitals, substantially weakening the stable N≡N triple bond and facilitating its subsequent reduction. Further insight was gained from the density of states (DOS) analysis (Figure 4b). Notably, the Ti_3_C_2_Cl_x_/Ti_3_ZnC_2_ heterostructure exhibits a high intensity of occupied states near the Fermi level, in contrast to the noticeable bandgap in Ti_3_C_2_Cl_x_. The metallic character of the heterostructure ensures charge carriers readily available for donation to N_2_ molecule, thereby promoting both adsorption and activation kinetics.

The complete reaction pathway was systematically calculated to compare the catalytic efficiency. The intermediate adsorption models show that the N‐containing intermediates are adsorbed by four Ti sites located on the interface of Ti_3_C_2_Cl_x_/Ti_3_ZnC_2_ model, compared to that of the three Ti sites on Ti_3_C_2_Cl_x_, suggesting a more robust and cooperative adsorption environment (Figures S24 and S25). The Gibbs free energy diagrams (Figure 4c) show that Ti_3_C_2_Cl_x_/Ti_3_ZnC_2_ and Ti_3_C_2_Cl_x_ demonstrate an identical rate‐determining step (RDS) of *NH_2_ converting to *NH_3_, while Ti_3_C_2_Cl_x_/Ti_3_ZnC_2_ heterostructure shows an RDS energy barrier of 0.7 eV, which is much lower than the 0.84 eV of Ti_3_C_2_Cl_x_. In addition, Ti_3_C_2_Cl_x_/Ti_3_ZnC_2_ heterostructure shows a more thermodynamically favored manner than Ti_3_C_2_Cl_x_ in the cleavage of the stable N─N bonds. In comparison, the alternating catalytic route of NRR was also performed on Ti_3_C_2_Cl_x_/Ti_3_ZnC_2_ and Ti_3_C_2_Cl_x_ models, where the *NNH_2_, *N, and *NH intermediates in the distal route were replaced by the *NHNH, *NHNH_2_, and *NH_2_NH_2_ intermediates, respectively (Figures S26 and S27). Ti_3_C_2_Cl_x_/Ti_3_ZnC_2_ in the alternating route shows the same RDS step to the distal route with the uphill energy barrier of 0.7 eV, whereas Ti_3_C_2_Cl_x_ shows a RDS step of *NHNH_2_ convert to *NH_2_NH_2_ with the much uphill energy barrier of 1.23 eV in the alternating route (Figure S28). Moreover, Ti_3_C_2_Cl_x_/Ti_3_ZnC_2_ also shows a more thermodynamic favored manner than Ti_3_C_2_Cl_x_ in breaking the N─N bonds of the critical *NH_2_NH_2_ intermediate in the alternating route. These results confirm that the Ti_3_C_2_Cl_x_/Ti_3_ZnC_2_ heterostructure exhibits superior NRR activity compared to Ti_3_C_2_Cl_x_. The NRR process on Ti_3_C_2_Cl_x_/Ti_3_ZnC_2_ follows the energetically more favorable distal pathway rather than the competitive alternating pathway (Figure 4d). In the distal pathway, Ti_3_C_2_Cl_x_/Ti_3_ZnC_2_ facilitates accelerated conversion of *NH_2_ intermediates to *NH_3_. The enhanced NRR activity of Ti_3_C_2_Cl_x_/Ti_3_ZnC_2_ heterostructure stems from the synergistic effects of the heterointerface, which creates a unique electronic structure. This structure exhibits optimized interfacial Ti sites for adsorption coupled with efficient electron transfer across the integrated system, thereby enabling profound N_2_ activation, stabilizing intermediates, and significantly reducing the kinetic barrier of the rate‐determining step.

### 2.4 Zn‐N_2_ Battery

**FIGURE 5:**
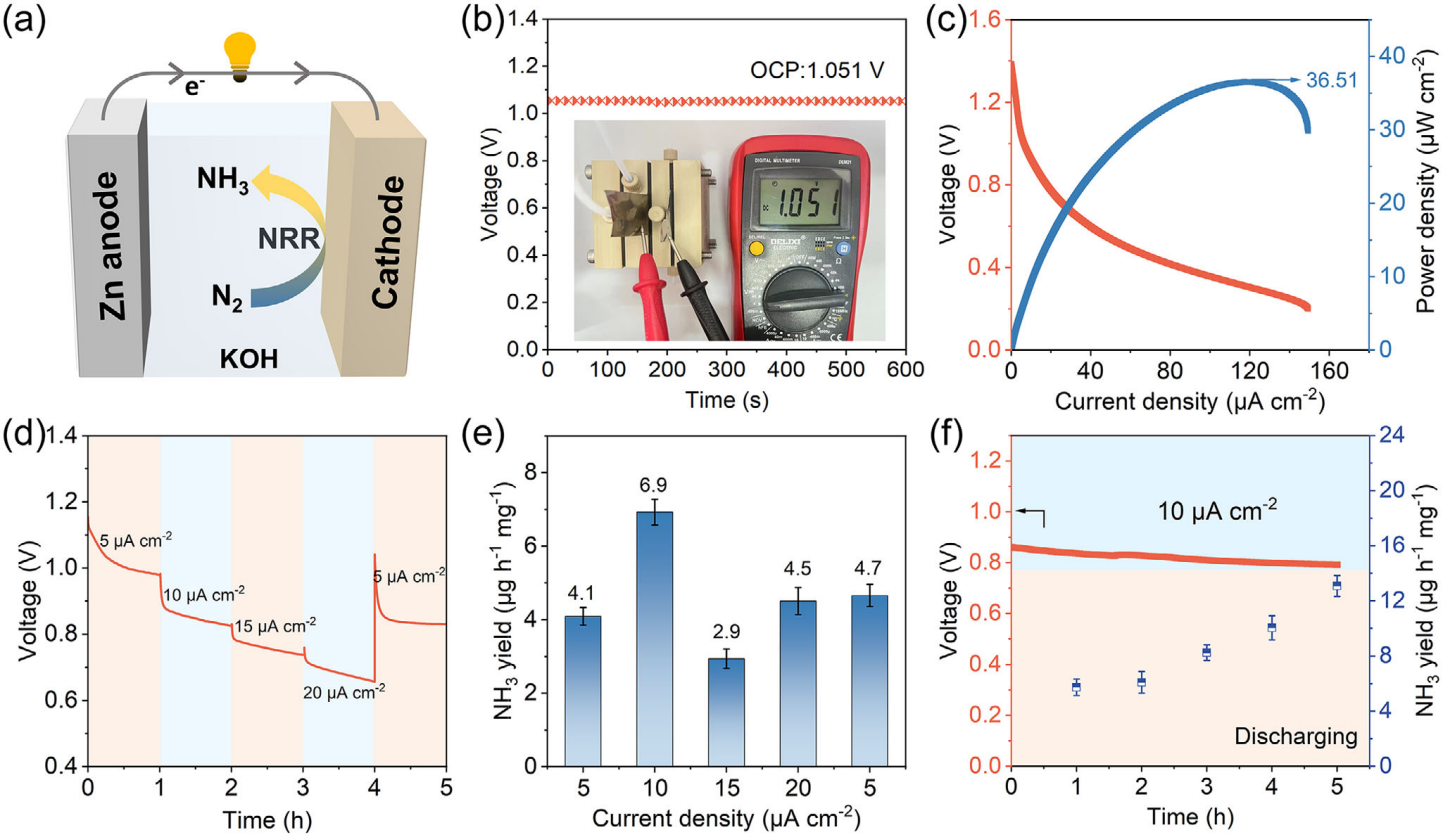
(a) Schematic of the Zn‐N_2_ battery. (b) OCV measured by CHI 760E and a multimeter. (c) Polarization curves and power density. (d) Discharging test and (e) NH_3_ yields at selected current densities. (f) The discharge curve at 10 µA cm^−2^ for continuous 5 h and NH_3_ yields for each 1 h.

The exceptional NRR activity of the Ti_3_C_2_Cl_x_/Ti_3_ZnC_2_ heterostructure makes it an ideal electrode for an aqueous Zn‐N_2_ battery for the co‐generation of NH_3_ and electricity. As a proof‐of‐concept experiment, an aqueous Zn‐N_2_ battery was constructed in 1 m KOH electrolyte with Ti_3_C_2_Cl_x_/Ti_3_ZnC_2_ as the cathode catalyst and a Zn foil as the anode (Figure 5a). The constructed Zn‐N_2_ battery delivers a stable open‐circuit voltage (OCV) of 1.051 V vs. Zn^2+^/Zn, in agreement with the data measured by the multimeter (Figure 5b). As shown in the polarization curve (Figure 5c), the Ti_3_C_2_Cl_x_/Ti_3_ZnC_2_‐based Zn‐N_2_ battery delivers a peak power density of 36.5 µW cm^−2^ at a current density of 119.3 µA cm^−2^, which not only exceeds that of Cu‐based (10.1 µW cm^−2^) [48] and VN@NSC‐based Zn‐N_2_ battery (16.4 µW cm^−2^) [49], but also surpasses that of some recently reported Zn‐N_2_ batteries (Table S2), underscoring the advantage of the heterostructure design. The practical operation of the battery was evaluated under various discharge conditions. It displays a stable discharging plateau at different current densities (5, 10, 15, and 20 µA cm^−2^) (Figure 5d). Importantly, both the voltage and NH_3_ yield return to initial levels when the current density was restored to 5 µA cm^−2^, demonstrating superior rate performance and electrochemical reversibility. During the discharge at 10 µA cm^−2^, the Ti_3_C_2_Cl_x_/Ti_3_ZnC_2_‐based battery obtains an NH_3_ yield of 6.9 µg h^−1^ mg^−1^ (Figure 5e). Additionally, the durability of the battery was further validated by a long‐term discharge test at 10 µA cm^−2^ (Figure 5f). After 5 h of continuous operation, the battery delivers an impressive NH_3_ yield of 13.1 µg h^−1^ mg^−1^. The XRD results (Figure S29) show no significant structural degradation, suggesting the structural and catalytic stability of the Ti_3_C_2_Cl_x_/Ti_3_ZnC_2_ cathode under working conditions. These integrated results demonstrate that the Ti_3_C_2_Cl_x_/Ti_3_ZnC_2_‐based Zn‐N_2_ battery is a highly promising system for simultaneous electrical power and electrochemical NH_3_ production, showcasing a novel route for distributed and sustainable nitrogen utilization.

## 3 Conclusions

In summary, we have successfully fabricated a Cl‐terminated Ti_3_C_2_Cl_x_/Ti_3_ZnC_2_ heterostructure via a dynamic etching approach, which functions as a highly efficient and robust electrocatalyst for ambient N_2_‐to‐NH_3_ conversion. The Ti_3_C_2_Cl_x_/Ti_3_ZnC_2_ heterostructure shows an outstanding NRR electrocatalytic capacity, with an NH_3_ yield of 20.1 µg h^−1^ mg^−1^ and a FE of 38.1% at −0.2 V vs. RHE in 0.1 m KOH, and a high stability for over 70 h. The high NRR performance of Ti_3_C_2_Cl_x_/Ti_3_ZnC_2_ heterostructure stems from the unique MXene/MAX heterostructure and the Cl‐terminated surface that modulates the electronic structure of interfacial Ti sites, optimizes the adsorption capacity of Ti for N‐containing intermediates, thereby reducing the energy barrier in the distal pathway. Meanwhile, enhanced oxidation resistance ensures structural stability during prolonged operation. The high conductivity of Ti_3_C_2_Cl_x_ further facilitates efficient electron transfer during N_2_ electroreduction. Additionally, a novel Zn‐N_2_ battery is fabricated by integrating a Ti_3_C_2_Cl_x_/Ti_3_ZnC_2_ heterostructure cathode with a Zn anode, which can deliver a peak power density of 36.5 µW cm^−2^ with an NH_3_ yield of 13.1 µg h^−1^ mg^−1^. Our work offers novel perspectives on constructing MXene‐based electrocatalysts through a dual‐modification strategy, as well as advancing Zn‐N_2_ batteries for simultaneous NH_3_ production and energy storage.

## Conflicts of Interest

The authors declare no conflict of interest.

## Supporting information




**Supporting File**: advs74217‐sup‐0001‐SuppMat.docx

## Data Availability

The data that support the findings of this study are available in the supplementary material of this article.
