# Supplementary material for: Dynamic Etching‐Induced Cl‐Terminated Ti3C2Clx/Ti3ZnC2 Heterostructure for Ammonia Electrosynthesis and Zinc‐Nitrogen Batteries
Source: Adv Sci (Weinh). 2026 Feb 4:e24280. Online ahead of print. doi: 10.1002/advs.202524280 (PMC13325665; doi:10.1002/advs.202524280)
Supplement: Supplementary file 1 — Supporting File: advs74217‐sup‐0001‐SuppMat.docx [file ADVS-9999-e24280-s001.docx]

Supporting Information

**Dynamic etching-induced Cl-terminated Ti_3_C_2_Cl_x_/Ti_3_ZnC_2_ heterostructure for ammonia electrosynthesis and zinc-nitrogen batteries**

*Yu Wang^1^, Ying Sun^1*^, Liqun Ye^2^, Jichi Liu^1^, Hui Li^3,4^, Yang Fu^3,4^, Fengzhan Sun^5^, Jieshan Qiu^6^, Chang Yu^7*^, Tianyi Ma^3,4*^*

*^1^ Institute of Clean Energy Chemistry, Key Laboratory for Green Synthesis and Preparative Chemistry of Advanced Materials of Liaoning Province, College of Chemistry, Liaoning University, Shenyang 110036, Liaoning, China*

*^2^ College of Materials and Chemical Engineering, Key Laboratory of inorganic nonmetallic crystalline and energy conversion materials, China Three Gorges University, Yichang 443002, China*

*^3^ Centre for Atomaterials and Nanomanufacturing (CAN), School of Science, RMIT University, Melbourne VIC 3000, Australia*

*^4^ ARC Industrial Transformation Research Hub for Intelligent Energy Efficiency in Future Protected Cropping (E2Crop), Australia*

*^5^ Shanghai Advanced Research Institute, Chinese Academy of Sciences, Shanghai 201210, P. R. China*

*^6^ College of Chemical Engineering, State Key Laboratory of Chemical Resource Engineering, Beijing University of Chemical Technology, Beijing 100029, China*

*^7^ State Key Laboratory of Fine Chemicals, Frontier Science Center for Smart Materials, Liaoning Key Lab for Energy Materials and Chemical Engineering, School of Chemical Engineering, Dalian University of Technology, Dalian, Liaoning 116024, China*

*Correspondence author*, e-mail: yingsun@lnu.edu.cn (Y. Sun), chang.yu@dlut.edu.cn (C. Yu) tianyi.ma@rmit.edu.au (T. Ma)*

**Experimental section**

**Chemicals and materials.** Ti_3_AlC_2_ powders (~300 mesh, 99.5%) was obtained from Laizhou KaiKai Ceramic Materials Co. Ltd. Nafion (5 wt%) was purchased from Shanghai Hesen Electric Co. Ltd. Zinc chloride (ZnCl_2_, AR), sodium hypochlorite solution (NaClO, available chlorine ≥ 5.0%), and ammonium chloride (NH_4_Cl, AR) were purchased from Aladdin Ltd. Concentrated hydrochloric acid (HCl, 36 wt.%), and potassium hydroxide (KOH, AR) were purchased from Shanghai Titan Scientific Co. Ltd. Sodium Salicylate (C_7_H_5_O_3_Na, AR), sodium nitroferricyanide (III) dehydrate (Na_2_Fe(CN)_5_NO·2H_2_O, AR), sodium potassium tartrate (NaKC_4_H_4_O_6_·4H_2_O, AR), ethanol (C_2_H_5_OH, AR), hydrazine hydrate (N_2_H_4_·H_2_O, 85%), concentrated sulfuric acid (H_2_SO_4_, 95%) and hydrogen peroxide aqueous solution (H_2_O_2_, 30.0%) were purchased from Sinopharma chemical reagent Co. Ltd. All the reagents were used as received without further purification. Nafion 117 membrane (Dupont) was purchased from the Fuelcell store. Ultrapure water used throughout all experiments was purified through a Millipore system (Millipore, 18.2 MΩ·cm). High purity N_2_ gas (≥ 99.999%) and Ar (≥ 99.999%) gas were bought from Shenyang Zhaote Gas Co. Ltd.

**Catalyst preparation.** The Ti_3_C_2_Cl_x_/Ti_3_ZnC_2_ heterostructure was obtained via Lewis acid molten salt etching route.^[1]^ In particular, Ti_3_AlC_2_ and ZnCl_2_ with a molar ratio of 1:6 were ground in an agate mortar. Then, the mixture was sintered at 600 ^o^C for 3 h in flowing Ar at a ramp rate of 5 ^o^C min^−1^, yielding the products that were washed with deionized water to remove the residual ZnCl_2_ and further soaked in a 3 M HCl solution under ultrasound for 1 h to remove Zn species. Then the products were dried at 40 ^o^C for 12 h in a vacuum oven, yielding the Ti_3_C_2_Cl_x_/Ti_3_ZnC_2_ heterostructure. The synthesis of Ti_3_ZnC_2_ and Ti_3_C_2_Cl_x_ catalysts was similar to that of Ti_3_C_2_Cl_x_/Ti_3_ZnC_2_ except that the reaction time was 1.5 and 5 h, respectively.

**Characterization**. SEM images were acquired using a Hitachi SU-8010 equipped with an EDS analyzer. TEM and HRTEM images were obtained on a JEM-2100 operating at 200 kV. XRD patterns were collected using a D8 Advance (Bruker) X-ray diffraction system with Cu Kα radiation (λ = 0.15406 nm). XPS spectra were recorded by the Thermo Scientific ESCALAB 250Xi spectrometer equipped with a monochromatic Al Kα X-ray source (1486.6 eV). The UV-vis spectra were obtained on a Shimadzu UV-2600 spectrophotometer.

**Electrochemical measurements.** Electrochemical data were collected with an electrochemical workstation (CHI 760E). Nafion 117 membranes were heated in 5% H_2_O_2_, 0.5 M H_2_SO_4_, and deionized water for 1 h, respectively, then rinsed thoroughly with deionized water. The membranes were immersed in deionized water for later use. Typically, 5 mg of the as-prepared catalysts and 20 μL of Nafion solution (5 wt%) were dispersed in 980 μL mixed solution of ethanol and H_2_O (v : v = 2 : 1) for 1 h sonication to form a homogeneous ink. Then, 20 µL catalyst ink was loaded on a 1 × 1 cm^2^ carbon paper (CP) and dried under ambient conditions for measurement. The catalyst-loaded CP was used as the working electrode, a platinum foil as the counter electrode, and the Hg/HgO electrode as the reference. All experiments were performed under ambient conditions. The volume of the electrolyte in the anode and cathode chambers is 20 mL. Before the NRR measurements, the 0.1 M KOH electrolyte was bubbled with high-purity N_2_ for 30 min and then bubbled at a constant flow rate of 20 mL min^−1^ throughout the whole electrolysis process. All potentials reported in this work were calibrated to reversible hydrogen electrode (RHE) according to the Nernst Equation: *E*_RHE_ = *E*_Hg/HgO_ + 0.059 × pH + 0.098, where *E*_Hg/HgO_ is the potential experimentally measured against the Hg/HgO reference electrode.

**Electrochemical Zn-N_2_ Battery Measurements.** The Zn-N_2_ battery performance tests were carried out on a homemade Zn-N_2_ battery filled with of 1 M KOH, where a polished Zn plate was used as an anode and carbon paper-supported catalyst as a cathode. The discharge product NH_3_ was absorbed by 0.01 M H_2_SO_4_. The discharge curve and galvanostatic tests of Zn-N_2_ battery were recorded on a CHI760E electrochemical workstation. The power density (*P*) of the Zn-N_2_ battery was determined by *P* = *I* × *V*, where *I* and *V* are the discharge current density and voltage, respectively.

**Standard solutions preparation.** First, 0.3146 g of NH_4_Cl (pretreat at 105 °C for 4 h) was immersed in 0.1 M KOH/0.01 M H_2_SO_4_ solution (100 mL) to obtain a 1000 μg_NH3_/mL standard solution. Then, 1 mL of 1000 μg _NH3_/mL standard solution was added into a 100 mL volumetric flask and added 0.1 M KOH/0.01 M H_2_SO_4_ solution to the scale mark to obtain a 10 μg _NH3_/mL standard solution. Finally, 0.0, 0.4, 0.8, 1.2, 1.6 and 2 mL of 10 μg _NH3_/mL stock solution were separately added into a 20 mL volumetric flask and added 0.10 M KOH/0.01 M H_2_SO_4_ solution to the scale mark to obtain 0.0, 0.2, 0.4, 0.6, 0.8, and 1.0 μg _NH3_/mL standard solutions.

**Determination of ammonia (NH_3_).** (a) The quantity of the produced NH_3_ was determined by indophenol blue method.^[2]^ Briefly, 4 mL of the standard solutions or post-electrolysis electrolyte was pipetted as well as 4 mL of 1 M NaOH solution containing 5 wt% salicylic acid and 5 wt% sodium citrate, followed by the addition of NaClO (0.05 M, 2 mL) solution and Na_2_Fe(CN)_5_NO·2H_2_O (1 wt%, 0.4 mL). After 1 h, the mixed solution was tested by ultraviolet-visible (UV-vis) spectrophotometer to obtain the absorption spectra. The formation of indophenol blue was determined using the absorbance at λ = 660 nm.

(b) Ammonia quantification by ammonia-sensitive selecting electrode method.^[3]^ First, a series of standard ammonia solutions (0.1, 0.2, 0.3, 0.4, and 0.5 µg_NH3_ mL^−1^ in 0.1 M KOH) were prepared from a stock solution (1000 ppm ammonia as nitrogen standard) for the calibration. And the electrode slope was checked for validity (slope should be between 54 and 60 in a temperature range of 20-25 °C). To minimize the impact of the background of the N_2_-saturated electrolyte (0.5 M NaOH) on the quantitative analysis of the produced NH_3_, each standard ammonia solution was prepared by the dilution of the stock NH_4_Cl solution using the N_2_-saturated 0.5 M NaOH solution. Ionic strength adjuster (ISA) was used to provide a constant background ionic strength and adjust the solution pH. ISA must be added to all samples and standards immediately before measurement to prevent ammonia loss, and 80 mL of standard or sample required the addition of 1.6 mL ISA with the stirring thoroughly. In addition, to test if ammonia escaped from the electrolyte solution, the outlet gas was introduced to an acid bottle for wet scrubbing to collect the possible escaping ammonia. The ion-selective electrode meter was employed to measure the ammonia concentration in the acid wet scrubbing bottle, and the experimental results revealed that no ammonia was detected, suggesting that the ammonia escaping from the electrolyte solution could be negligible.

**Determination of hydrazine (N_2_H_4_).** The possible hydrazine product in the electrolytes was estimated by Watt and Chrisp method.^[4]^ To prepare a sensitive chromogenic reagent, para-(dimethylamino) benzaldehyde (2.0 g) was dissolved in a mixture of HCl (10 mL, concentrated) and C_2_H_5_OH (100 mL). In detail, 5 mL electrolyte was added into 5 mL chromogenic reagent, and the adsorption spectrum was obtained by using UV-vis spectrophotometer after 15 min. The concentration of hydrazine was determined using the absorbance signal at λ = 455 nm.

**Calculations of NH_3_ yields (R_NH3_) and FE.** The ammonia formation rate was determined using the following Equation:

$\text{NH}\text{3}\text{ yield} = \text{(}\text{c}\text{ × }\text{V}\text{)/(}\text{t}\text{ × }\text{m}\text{)}$ (1)

where *c* is the measured NH_3_ concentration (mol L^−1^), *V* is the volume of the electrolyte (L), *t* is the reduction reaction time (h), and *m* is the mass of the catalyst (mg).

The Faradaic efficiency was calculated according to following Equation:

$\text{FE = (3 × }\text{F}\text{ × }\text{c}\text{ × }\text{V}\text{)/(17 × }\text{Q}\text{)}$ (2)

where *F* is the Faraday constant (96485 C mol^−1^), *c* is the measured NH_3_ concentration (mol L^−1^), *V* is the volume of the electrolyte (L), and *Q* is the total charge used for the electrodes.

**DFT calculations.** All calculations were performed using the spin-polarized density functional theory (DFT) method through Vienna Ab initio Simulation Package (VASP5.4.4).^[5,6]^ Generalized gradient approximation (GGA) of Perdew-Burke Ernzerhof (PBE) were adopted to describe exchange correlation interaction.^[7]^ The ion-electron interaction was treated using the projector augmented wave (PAW) technique.^[8]^ The plane-wave cutoff energy of 450 eV was employed. The atomic positions were fully relaxed until the maximum force on each atom was less than 0.02 eV/Å, and total energy convergence was achieved within 10^−5^ eV. The Brillouin zone was sampled using the Monkhorst-Pack scheme with a k-point mesh of 3 × 3 × 1 in the Γ-centered grids for the structural relaxation. The Ti 3d electrons were corrected using DFT + U method with an effective U value of 3.0 eV.


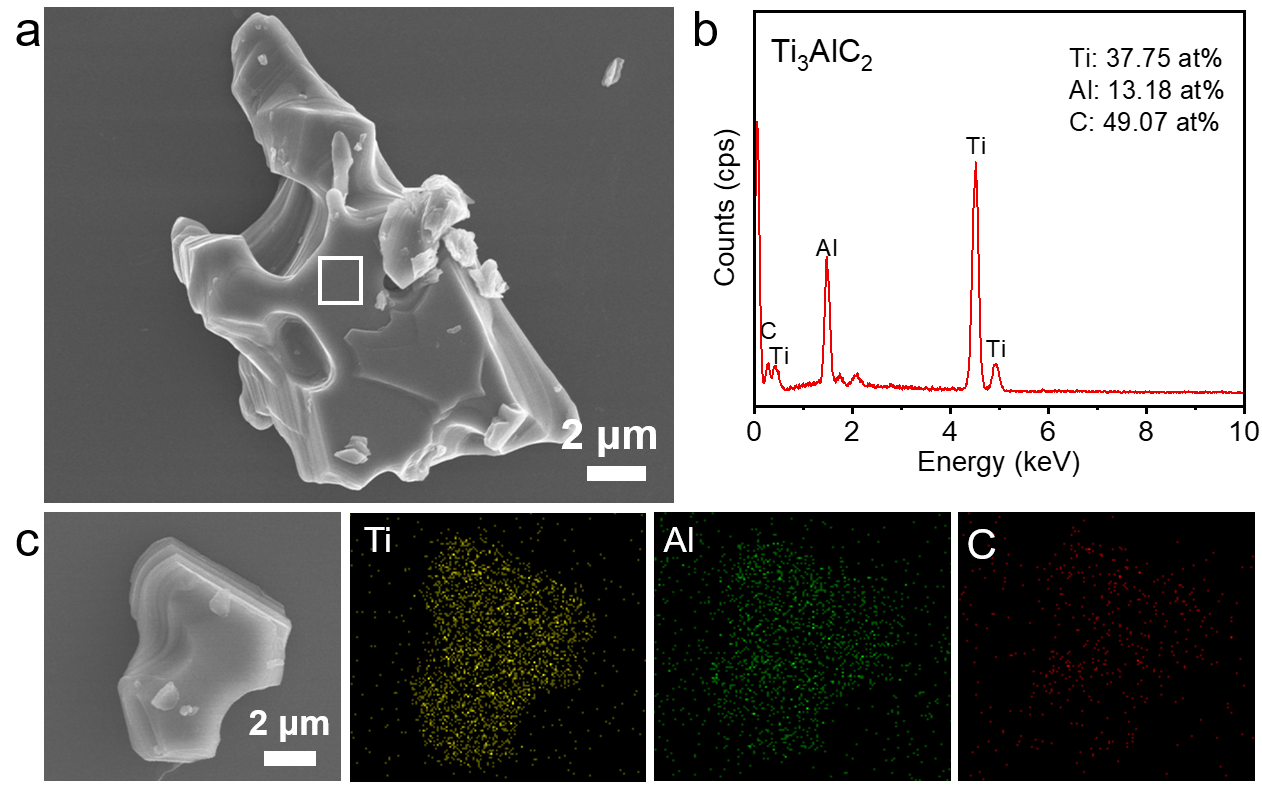


**Fig. S1** (a) SEM, (b) corresponding EDS analysis, and (c) element mapping of Ti_3_AlC_2_.


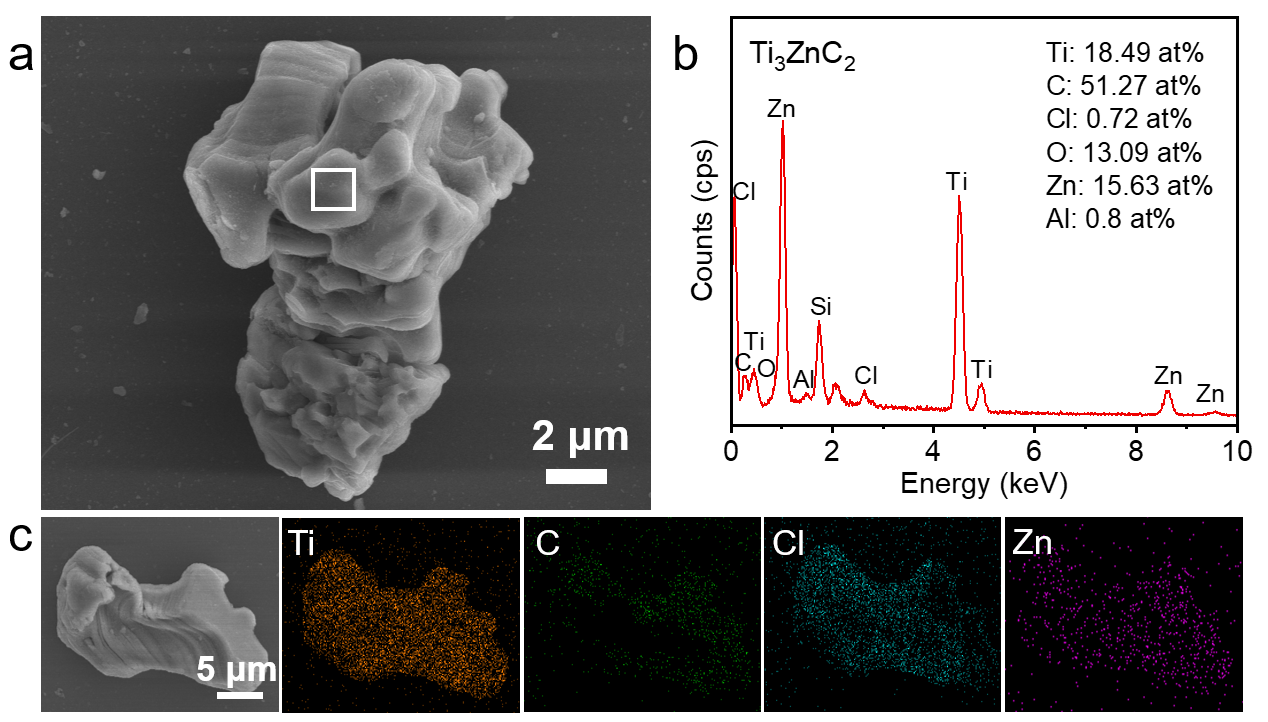


**Fig. S2** (a) SEM, (b) corresponding EDS analysis, and (c) element mapping of Ti_3_ZnC_2_.


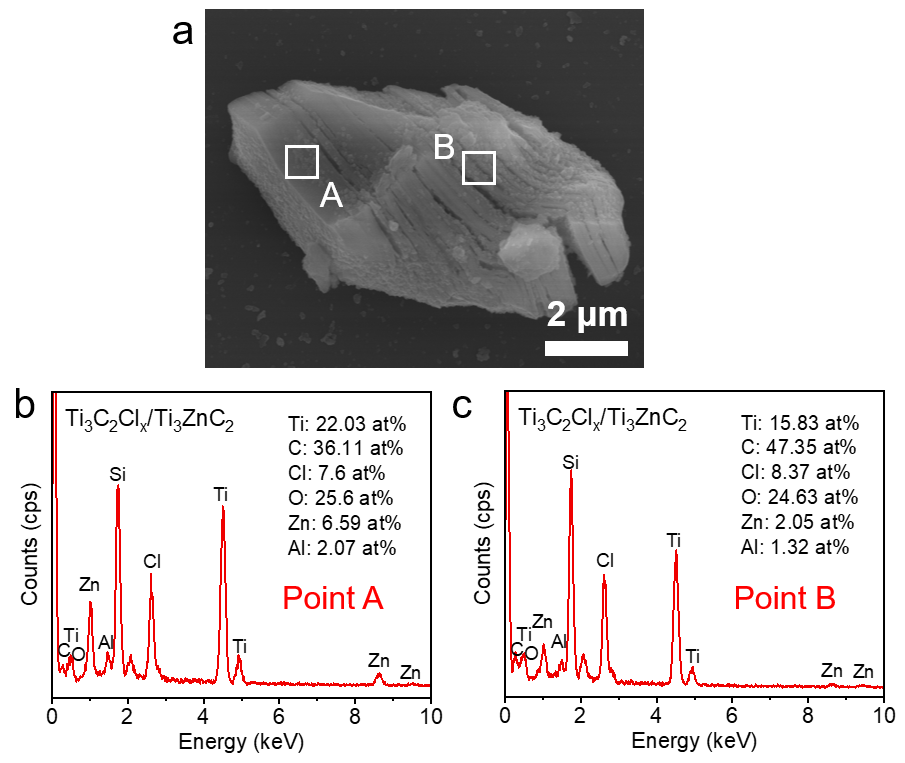


**Fig. S3** (a) SEM, and (b and c) corresponding EDS analysis of Ti_3_C_2_Cl_x_/Ti_3_ZnC_2_.

The composition ratio was estimated by inductively coupled plasma optical emission spectrometry (ICP-OES), which revealed a zinc mass of ca. 0.02 g in the 0.53 g Ti_3_C_2_Cl_x_/Ti_3_ZnC_2_ composite, corresponding to a Ti_3_C_2_Cl_x_/Ti_3_ZnC_2_ mass ratio of 6.6 : 1.


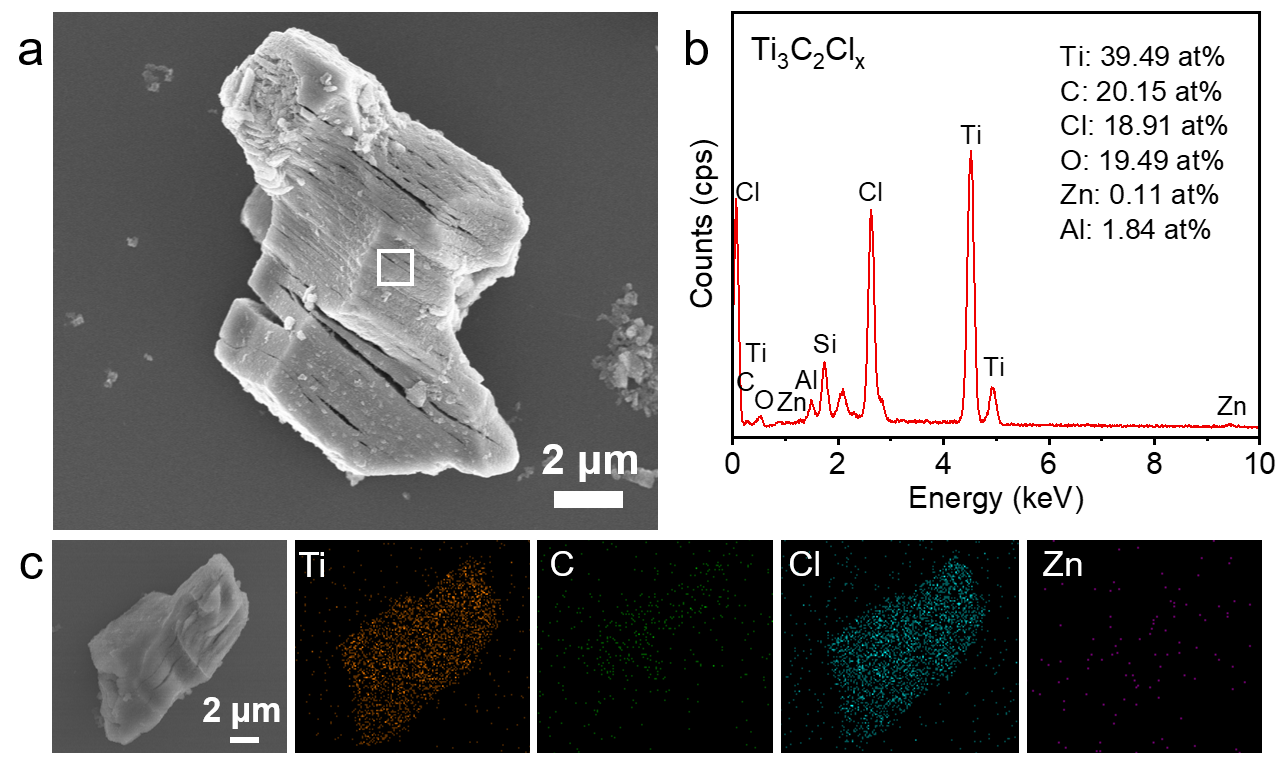


**Fig. S4** (a) SEM, (b) corresponding EDS analysis, and (c) element mapping of Ti_3_C_2_Cl_x_.


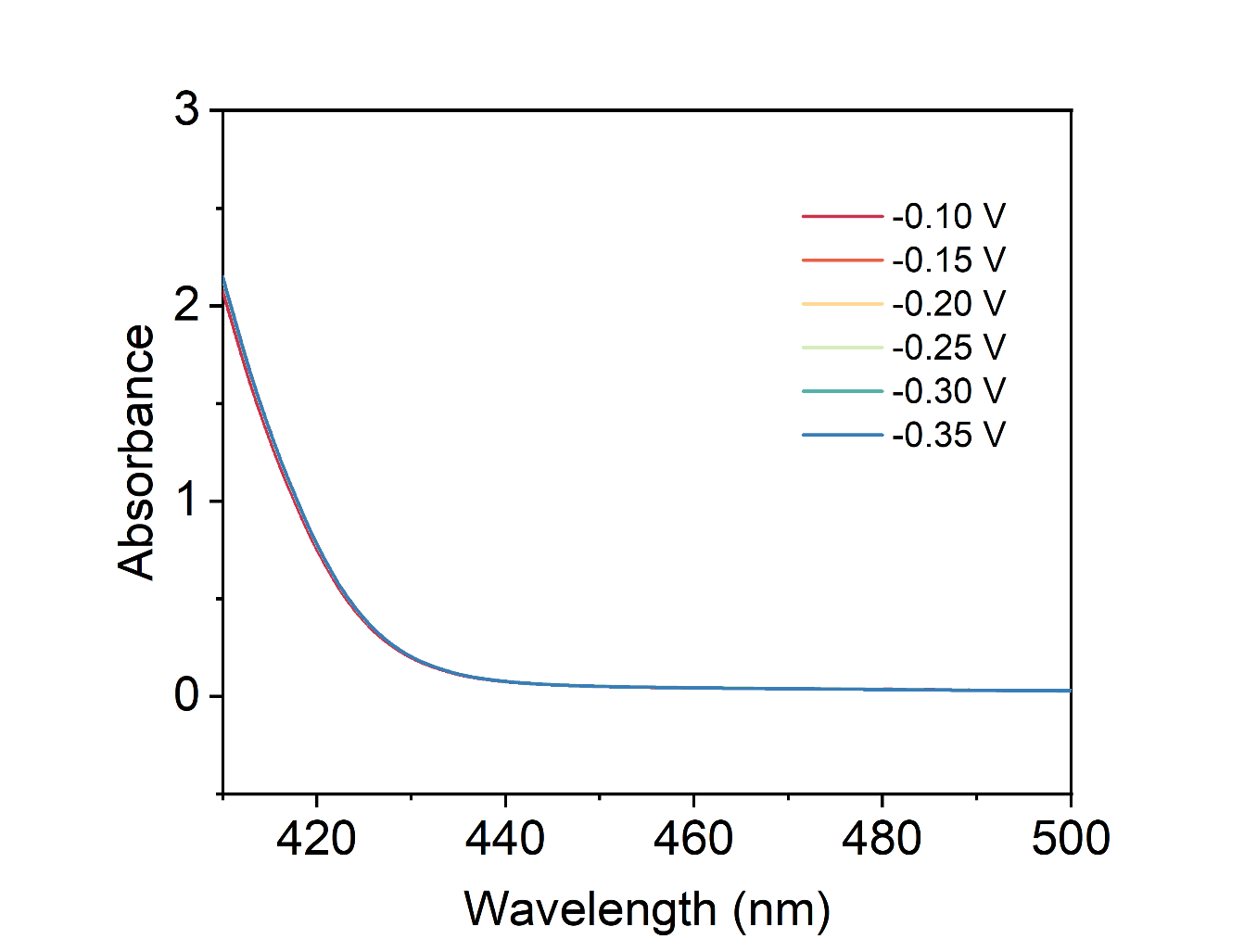


**Fig. S5** UV-vis absorption spectra of N_2_H_4_ generated by Ti_3_C_2_Cl_x_/Ti_3_ZnC_2_ at different applied potentials in 0.1 M KOH.


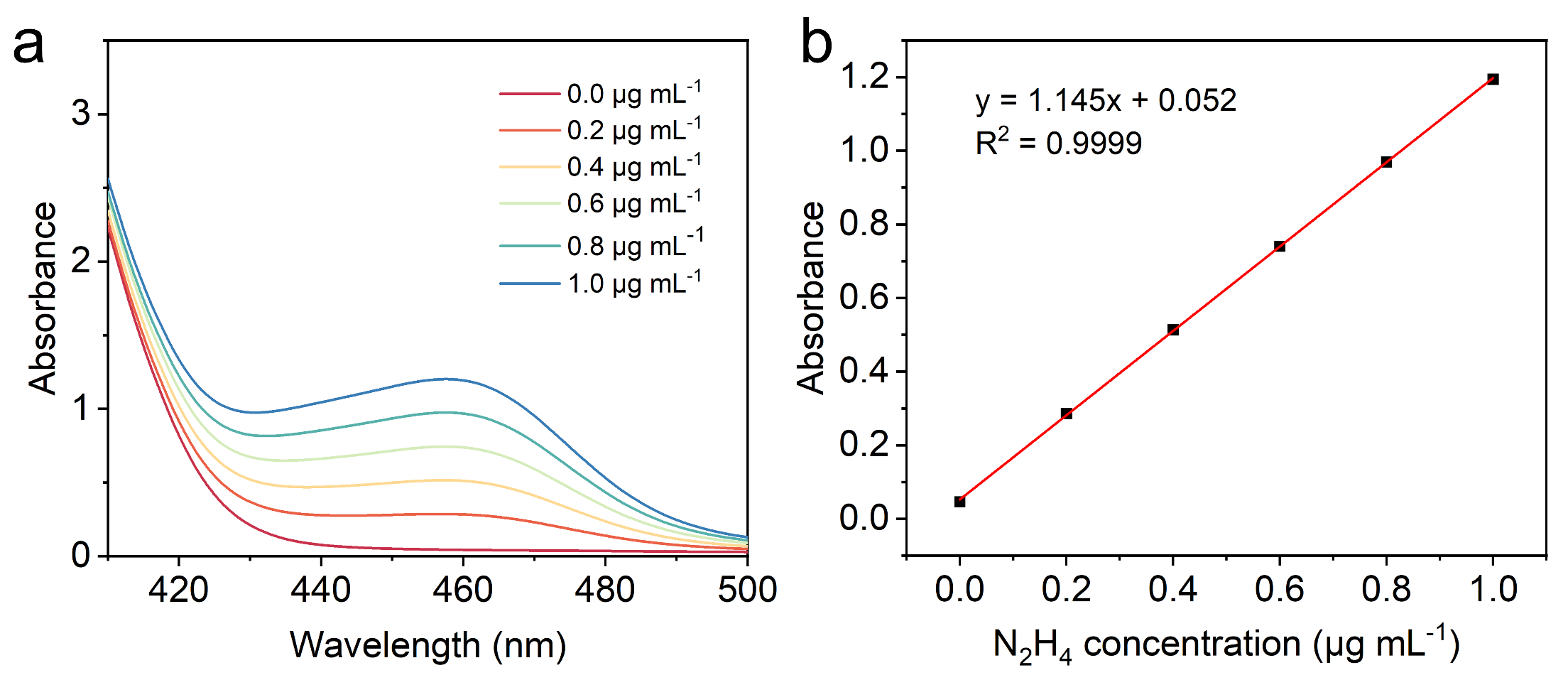


**Fig. S6** (a) UV-vis absorption spectra of standard solutions containing different concentrations of N_2_H_4_ in 0.1 M KOH. (b) Corresponding calibration curve.


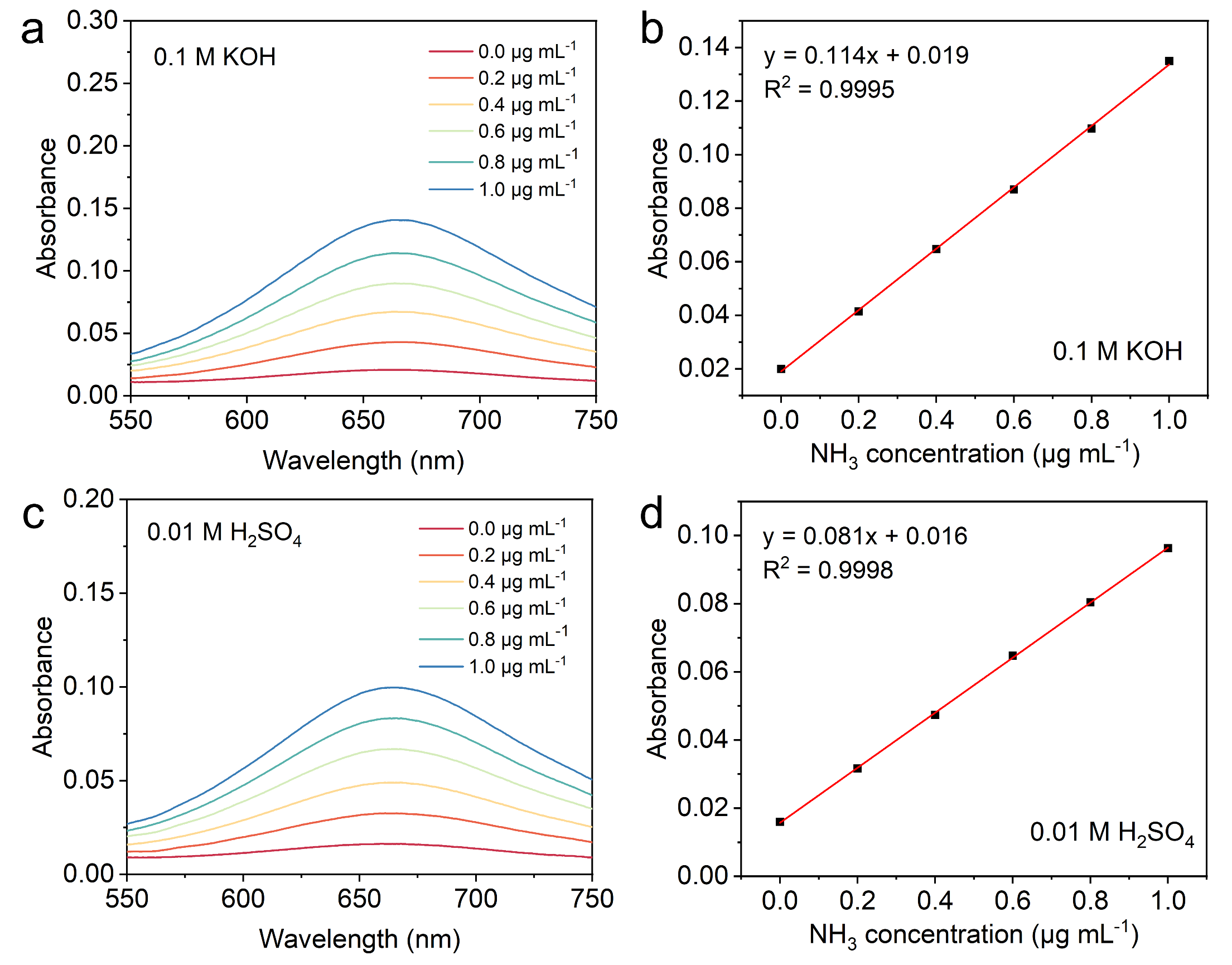


**Fig. S7** UV-vis absorption spectra for known-concentration (a) NH_4_Cl solution in 0.1 M KOH, (c) NH_4_Cl solution in 0.01 M H_2_SO_4_. Corresponding calibration curve in (b) 0.1 M KOH for NH_3_, (d) 0.01 M H_2_SO_4_ for NH_3_.


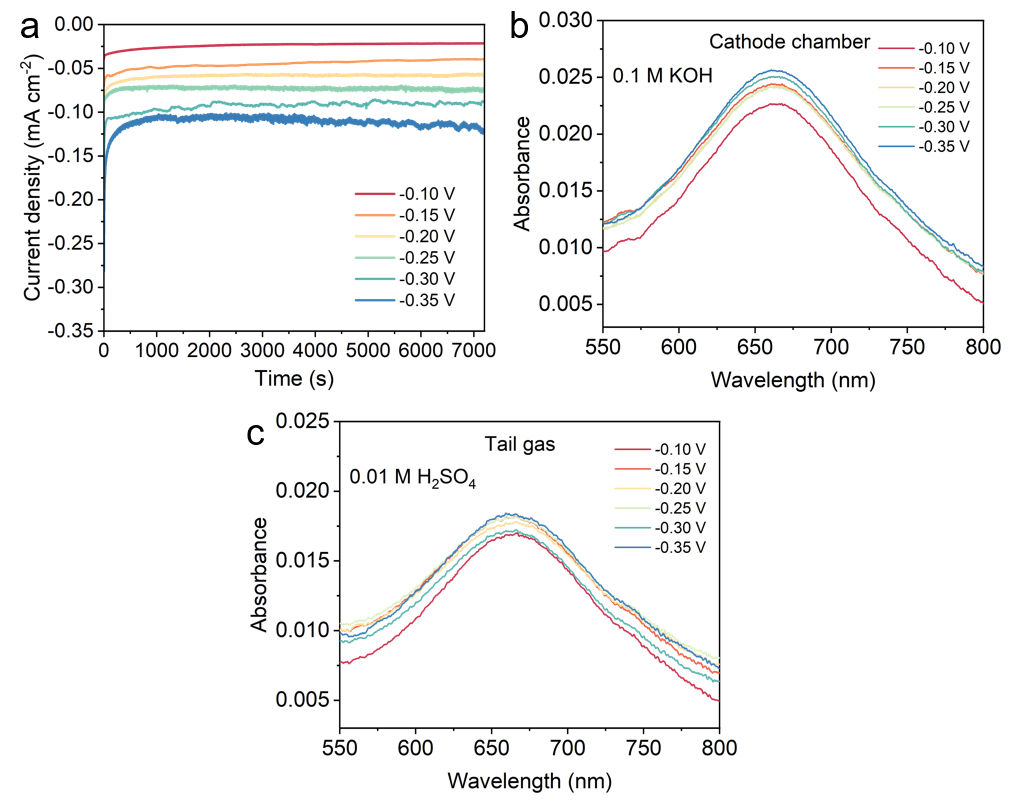


**Fig. S8** (a) Chronoamperometry curves of Ti_3_AlC_2_ at different applied potentials in N_2_-saturated 0.1 M KOH electrolyte. Corresponding UV-vis absorption spectra in (b) cathode chamber (0.1 M KOH), (c) tail gas (0.01 M H_2_SO_4_).


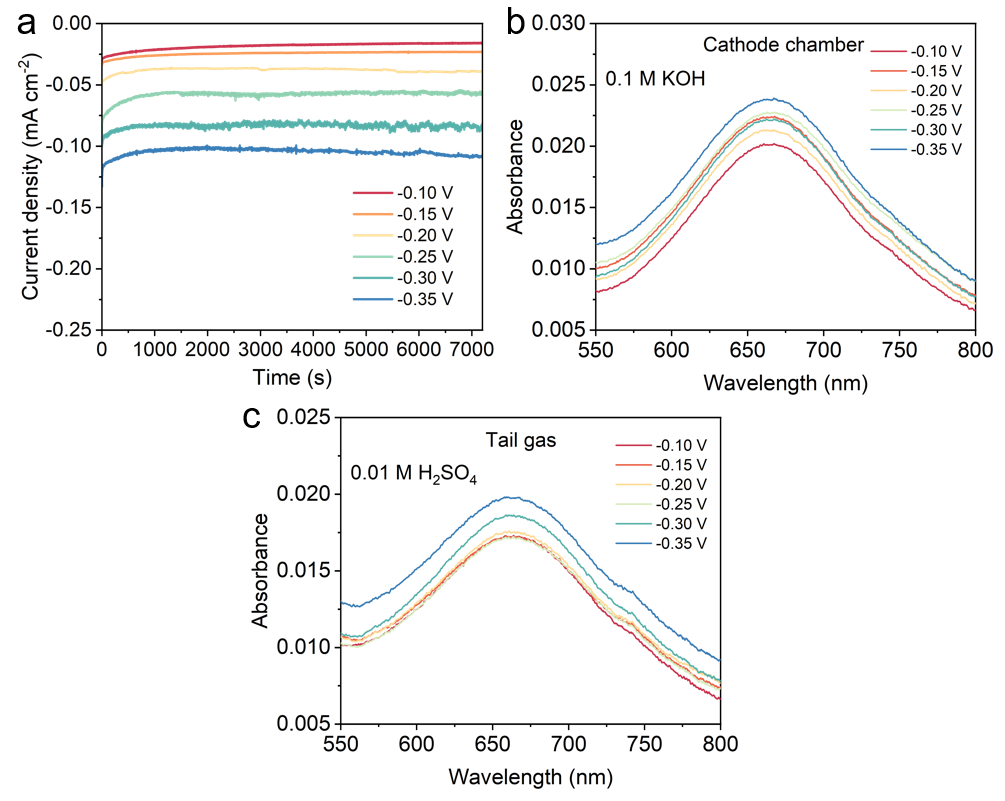


**Fig. S9** (a) Chronoamperometry curves of Ti_3_ZnC_2_ at different applied potentials in N_2_-saturated 0.1 M KOH electrolyte. Corresponding UV-vis absorption spectra in (b) cathode chamber (0.1 M KOH), (c) tail gas (0.01 M H_2_SO_4_).


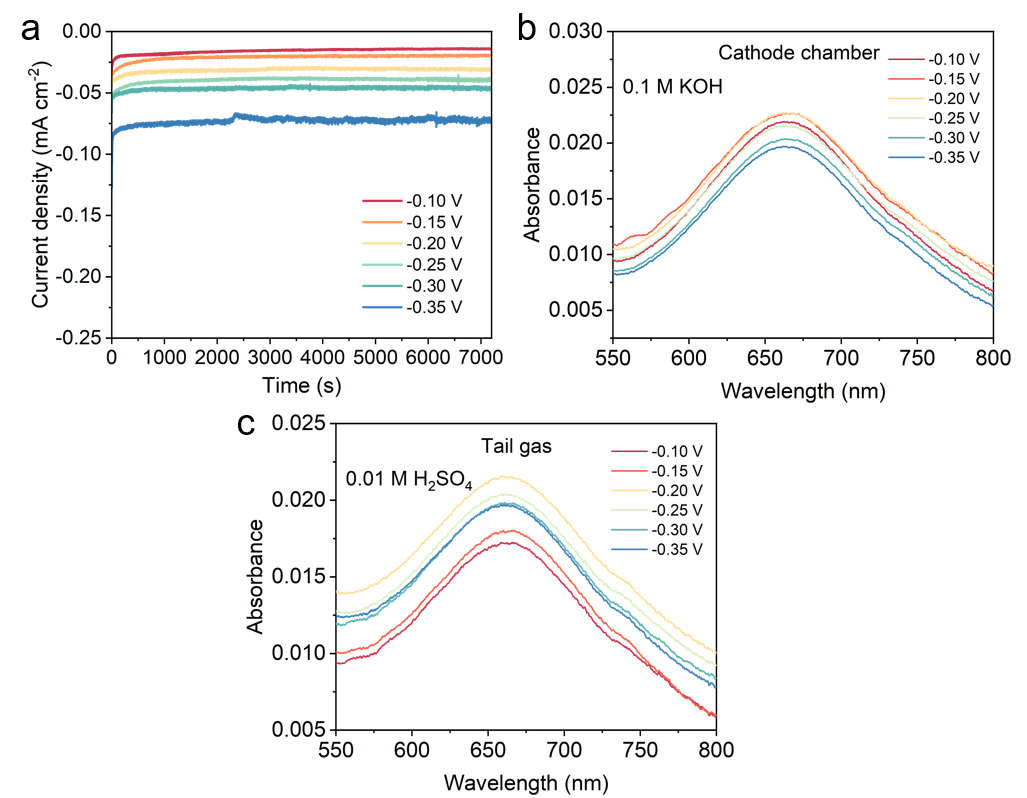


**Fig. S10** (a) Chronoamperometry curves of Ti_3_C_2_Cl_x_/Ti_3_ZnC_2_ at different applied potentials in N_2_-saturated 0.1 M KOH electrolyte. Corresponding UV-vis absorption spectra in (b) cathode chamber (0.1 M KOH), (c) tail gas (0.01 M H_2_SO_4_).


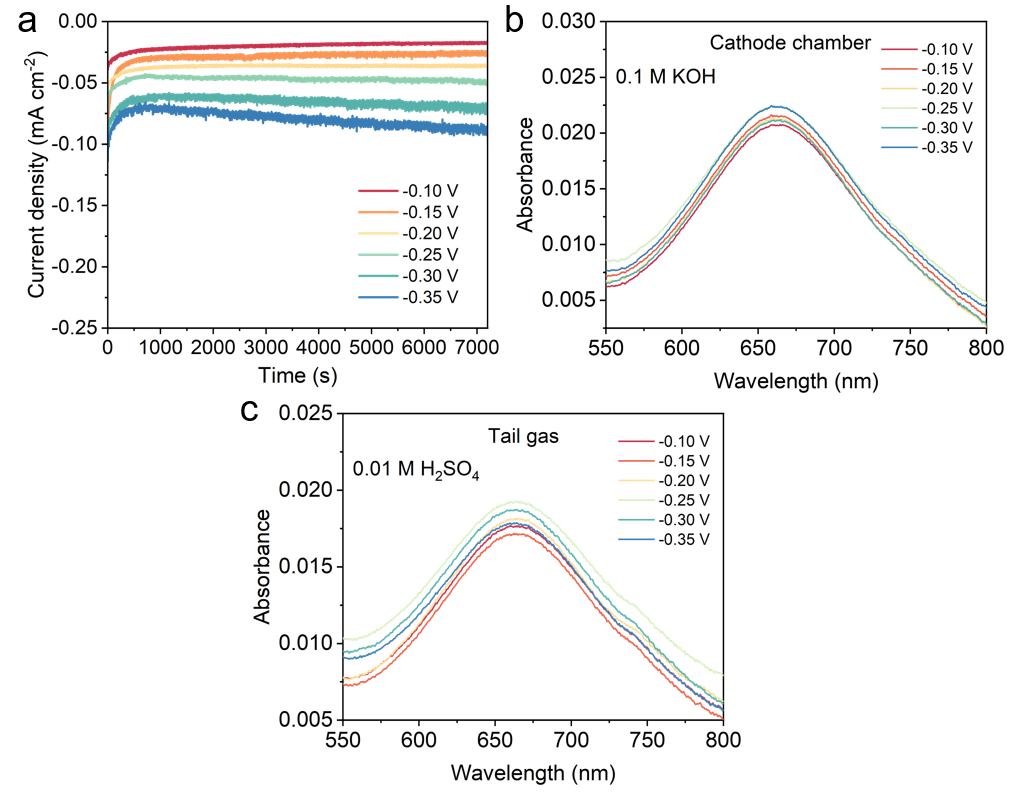


**Fig. S11** (a) Chronoamperometry curves of Ti_3_C_2_Cl_x_ at different applied potentials in N_2_-saturated 0.1 M KOH electrolyte. Corresponding UV-vis absorption spectra in (b) cathode chamber (0.1 M KOH), (c) tail gas (0.01 M H_2_SO_4_).


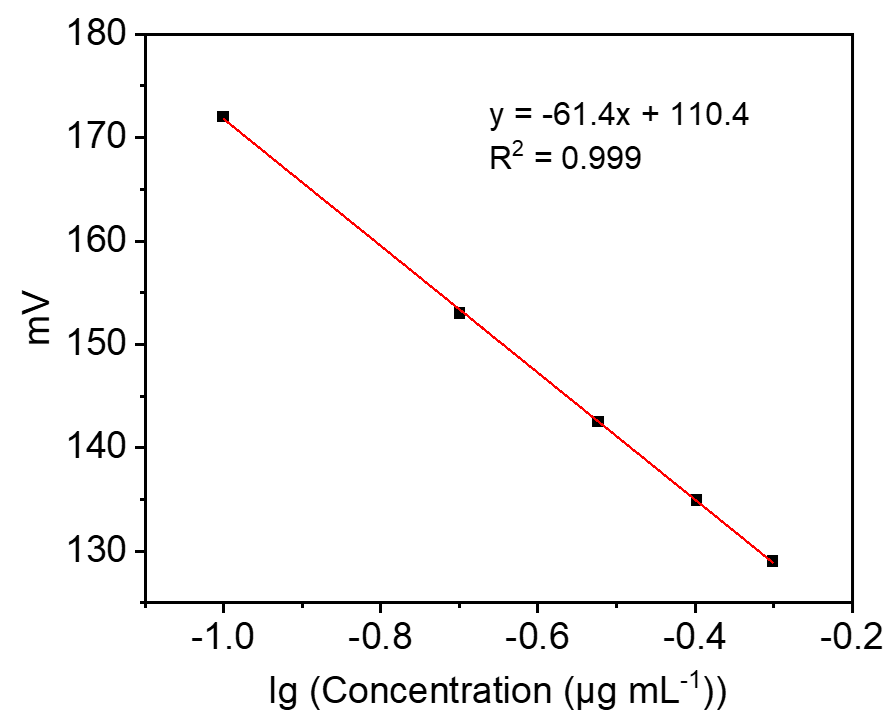


**Fig. S12** The standard curve for the standard NH_4_Cl solution with different content detected by the ammonia-sensitive electrode.


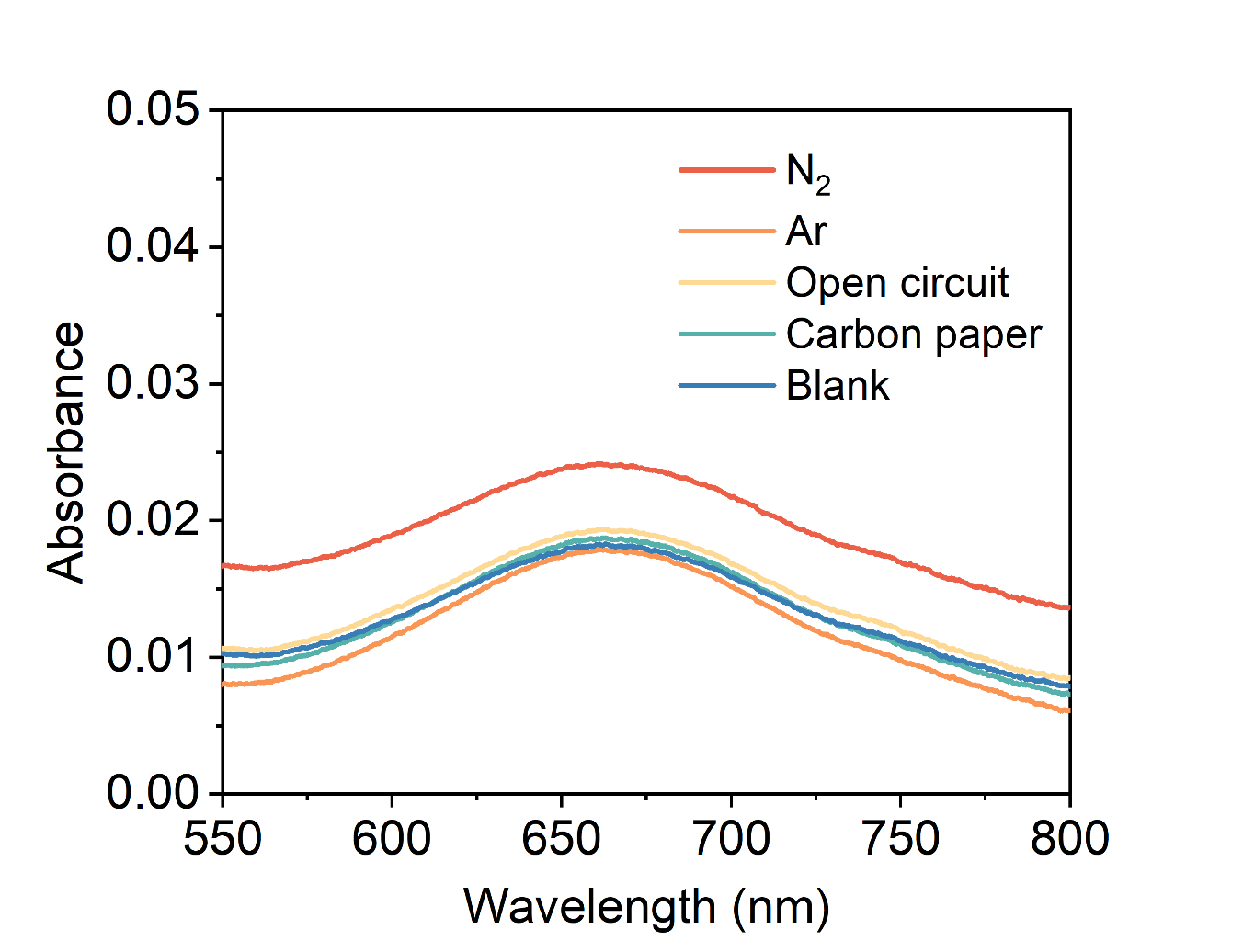


**Fig. S13** UV-vis absorption spectra of N_2_-saturated post electrolytes at −0.2 V vs. RHE (red), Ar-saturated post electrolytes at −0.2 V vs. RHE (orange), N_2_-saturated post electrolytes under open circuit potential (yellow), carbon paper as the working electrode under N_2_-saturated at −0.2 V vs. RHE (green), and blank 0.1 M KOH electrolyte (blue).

**
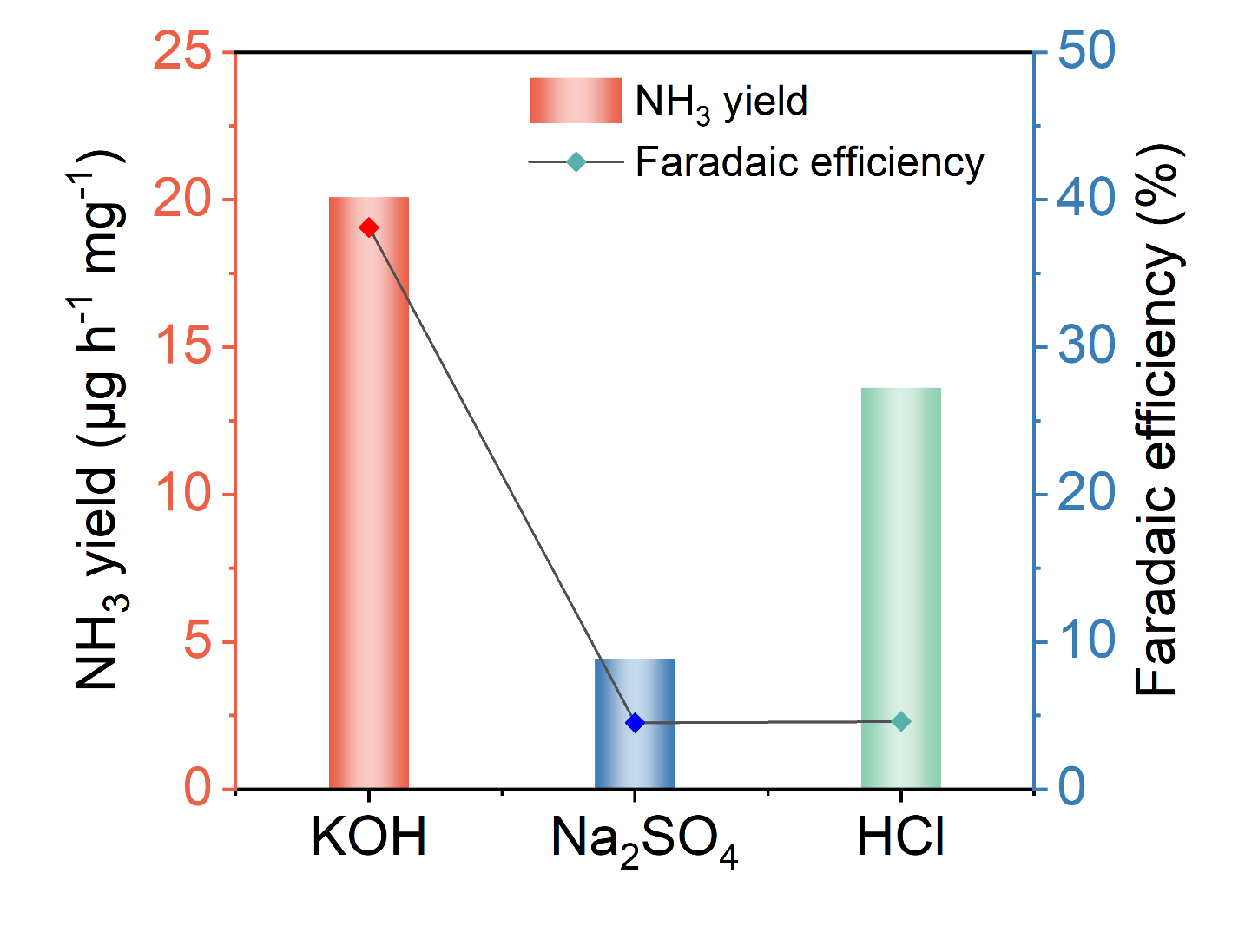
**

**Fig. S14** NH_3_ yields and FEs of Ti_3_C_2_Cl_x_/Ti_3_ZnC_2_ in various electrolytes.


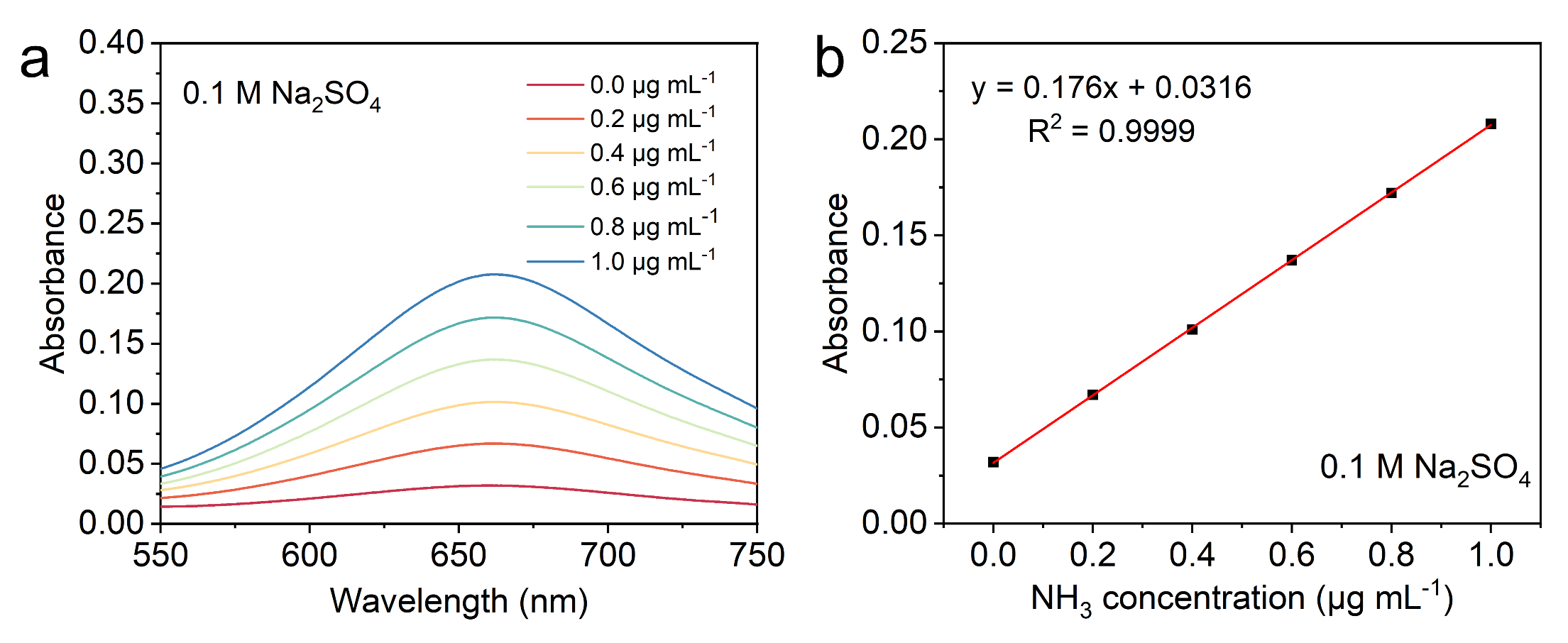


**Fig. S15** (a) UV-vis absorption spectra for known-concentration NH_4_Cl solution in 0.1 M Na_2_SO_4_. (b) Corresponding calibration curve in 0.1 M Na_2_SO_4_.


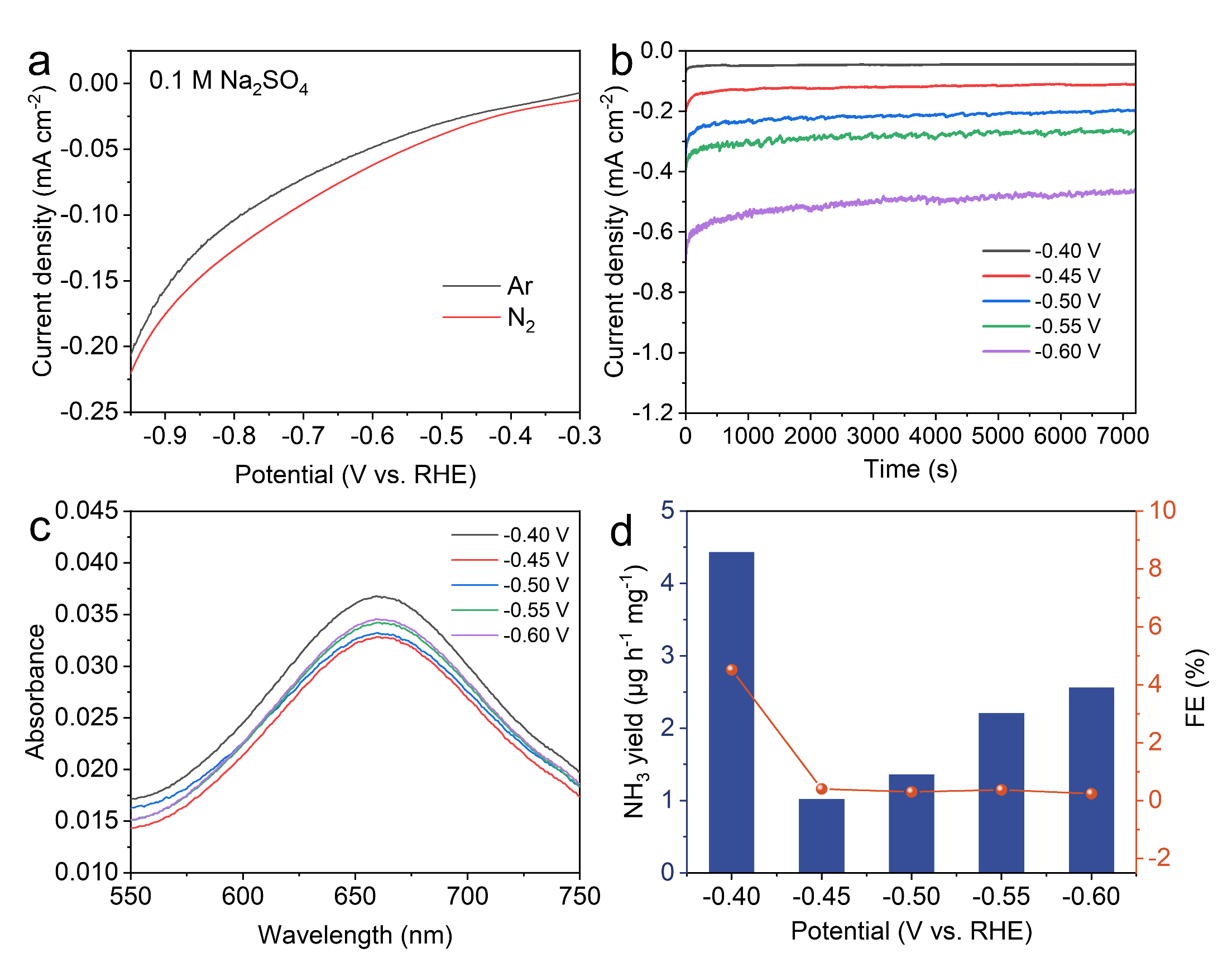


**Fig. S16** (a) LSV curves of Ti_3_C_2_Cl_x_/Ti_3_ZnC_2_ in the Ar- and N_2_-saturated 0.1 M Na_2_SO_4_ solutions. (b) Chronoamperometry curves under different applied potentials. (c) Corresponding UV-vis absorption spectra. (d) Corresponding NH_3_ yields and FEs.


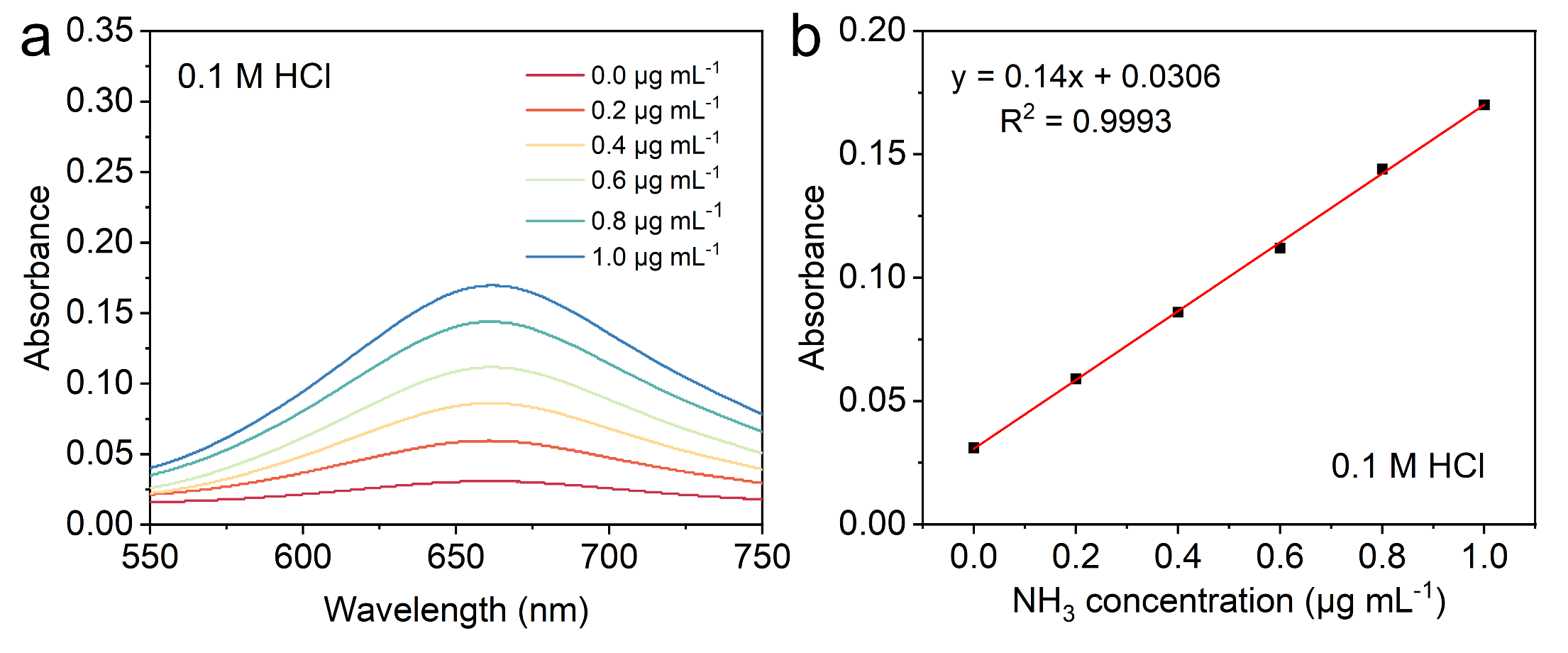


**Fig. S17** (a) UV-vis absorption spectra for known-concentration NH_4_Cl solution in 0.1 M HCl. (b) Corresponding calibration curve in 0.1 M HCl.


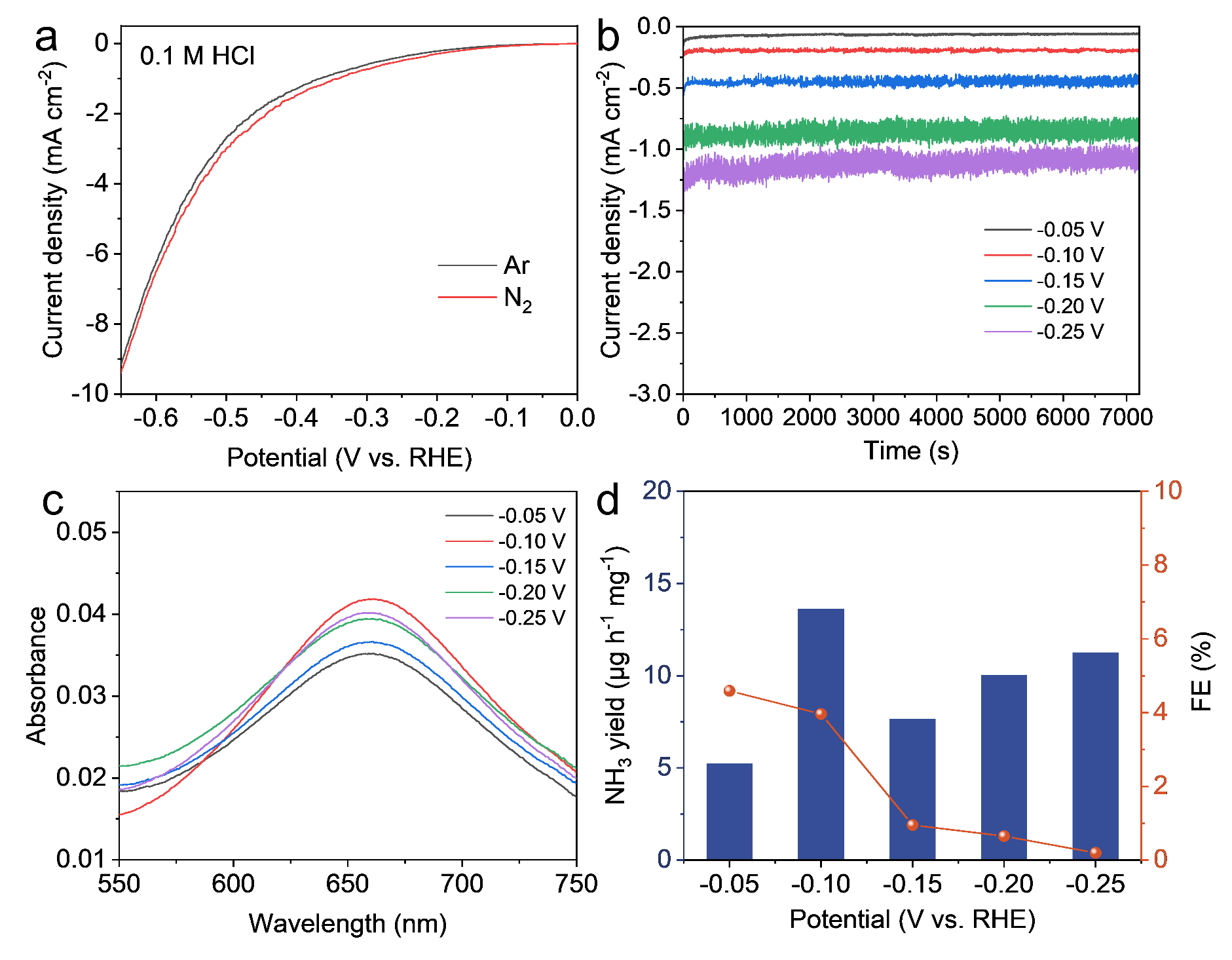


**Fig. S18** (a) LSV curves of Ti_3_C_2_Cl_x_/Ti_3_ZnC_2_ in the Ar- and N_2_-saturated 0.1 M HCl solutions. (b) Chronoamperometry curves under different applied potentials. (c) Corresponding UV-vis absorption spectra. (d) Corresponding NH_3_ yields and FEs.


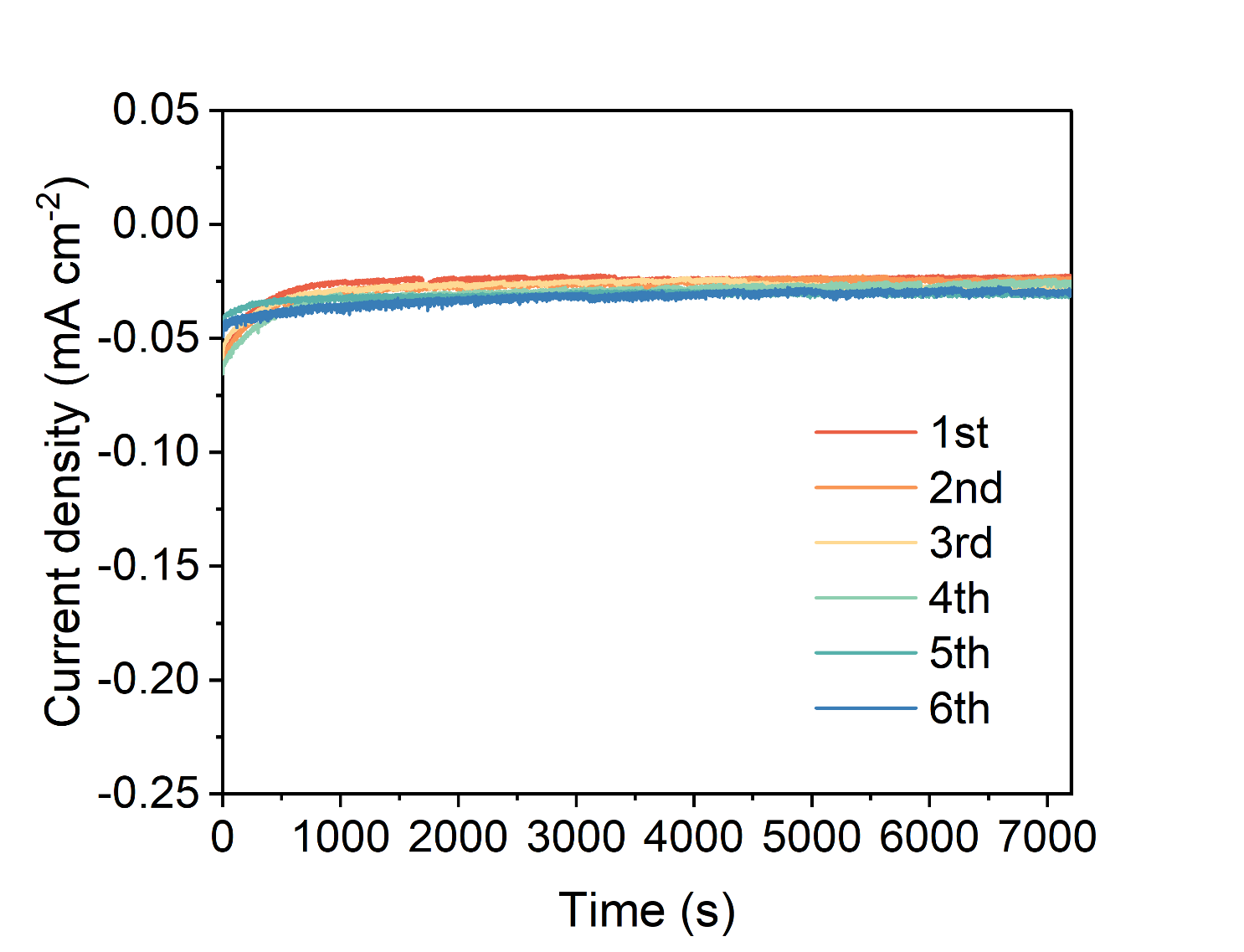


**Fig. S19** Chronoamperometry curves of NRR on Ti_3_C_2_Cl_x_/Ti_3_ZnC_2_ heterostructure at -0.2 V vs. RHE for 6 cycles.


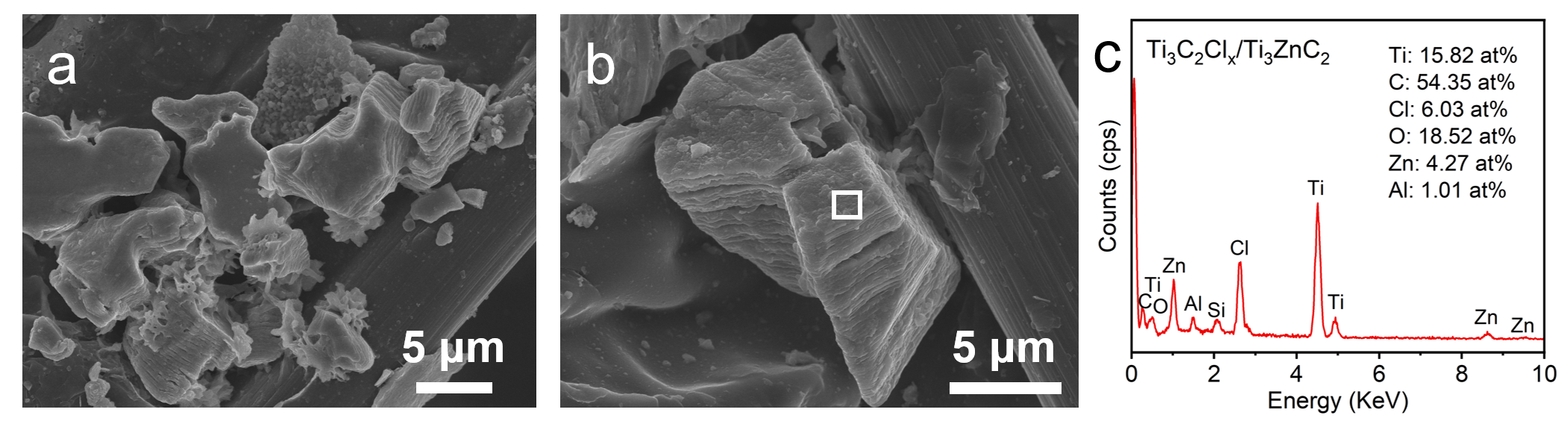


**Fig. S20** (a) SEM images and (b) corresponding EDS analysis of Ti_3_C_2_Cl_x_/Ti_3_ZnC_2_ heterostructure after stability test.

**
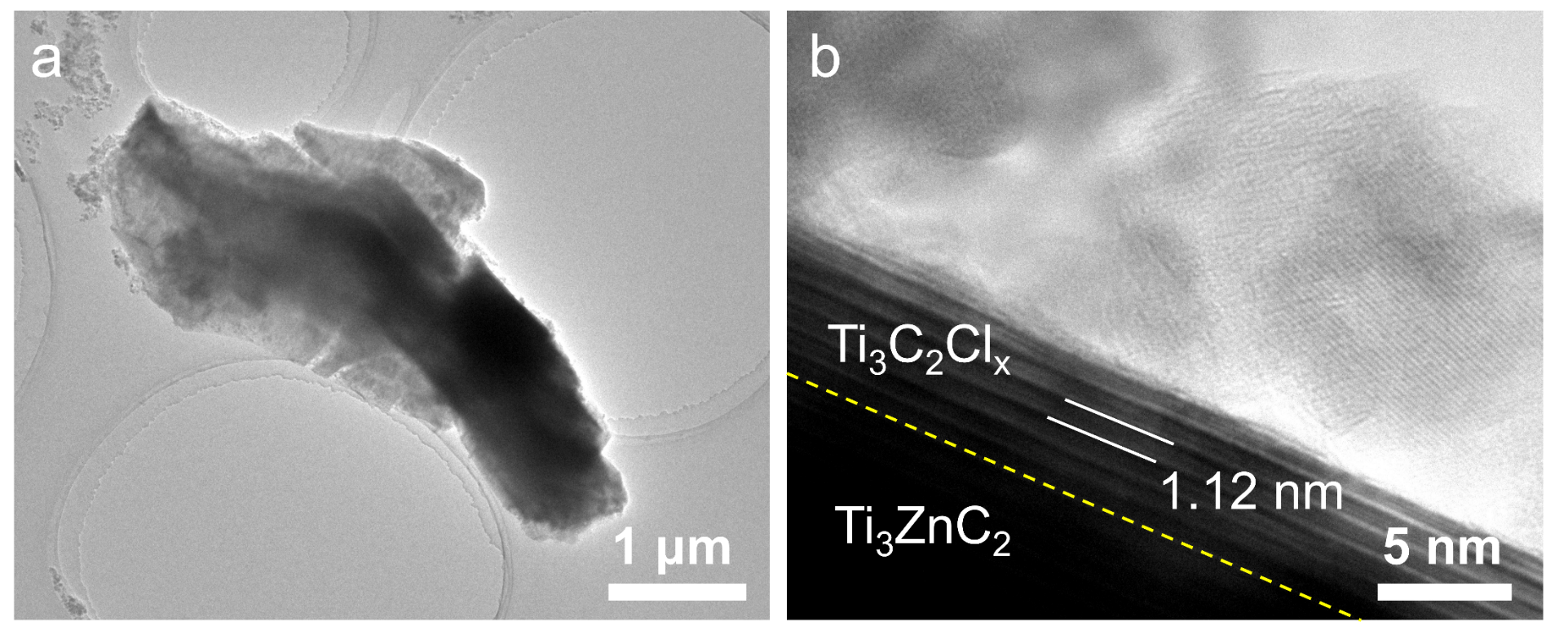
**

**Fig. S21** (a) TEM and (b) HRTEM images of Ti_3_C_2_Cl_x_/Ti_3_ZnC_2_ heterostructure after stability test.


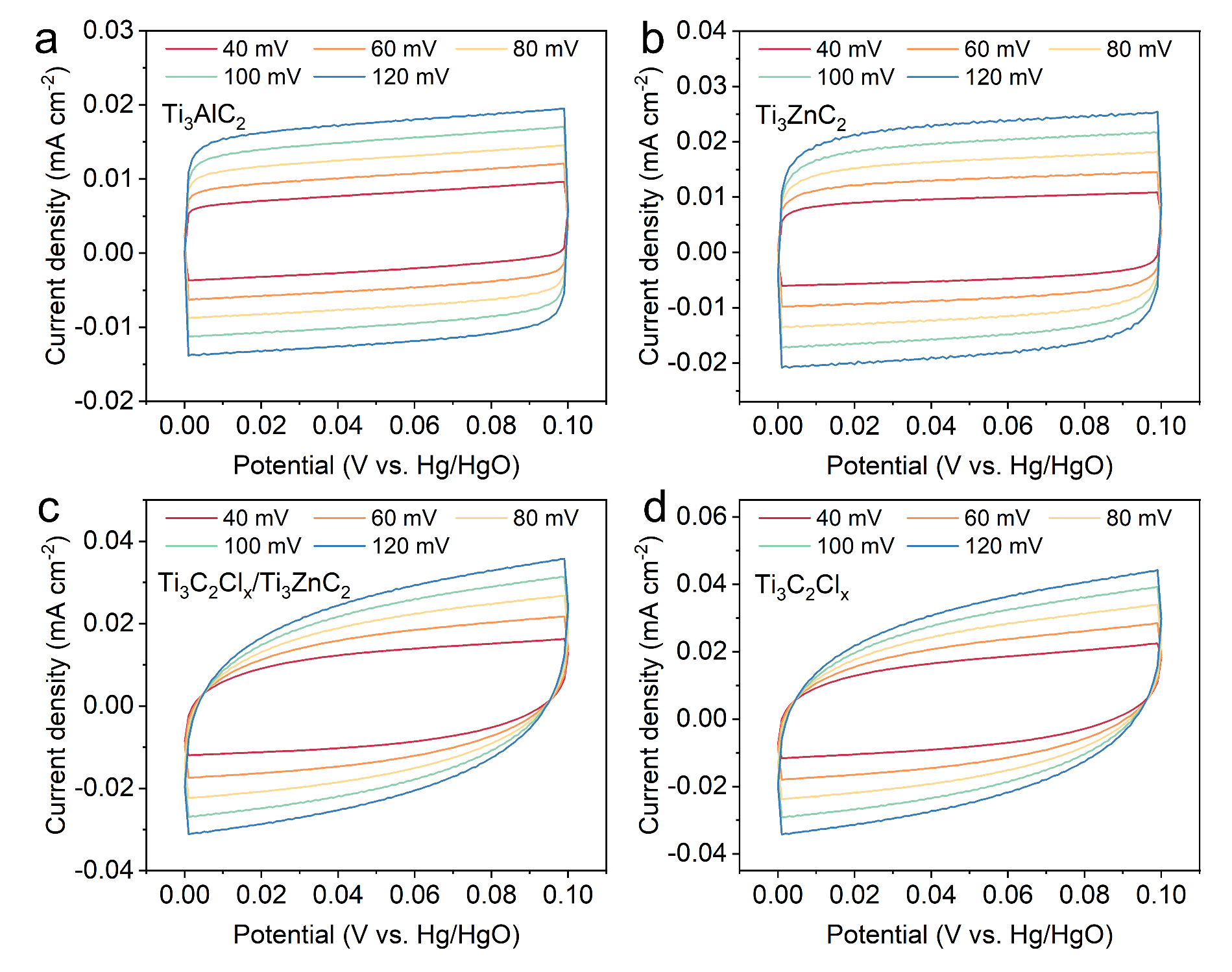


**Fig. S22** Cyclic voltammogram curves in the double layer region at the scan rate of 40, 60, 80, 100, 120 mV s^-1^ of (a) Ti_3_AlC_2_, (b) Ti_3_ZnC_2_, (c) Ti_3_C_2_Cl_x_/Ti_3_ZnC_2_, and (d) Ti_3_C_2_Cl_x_.


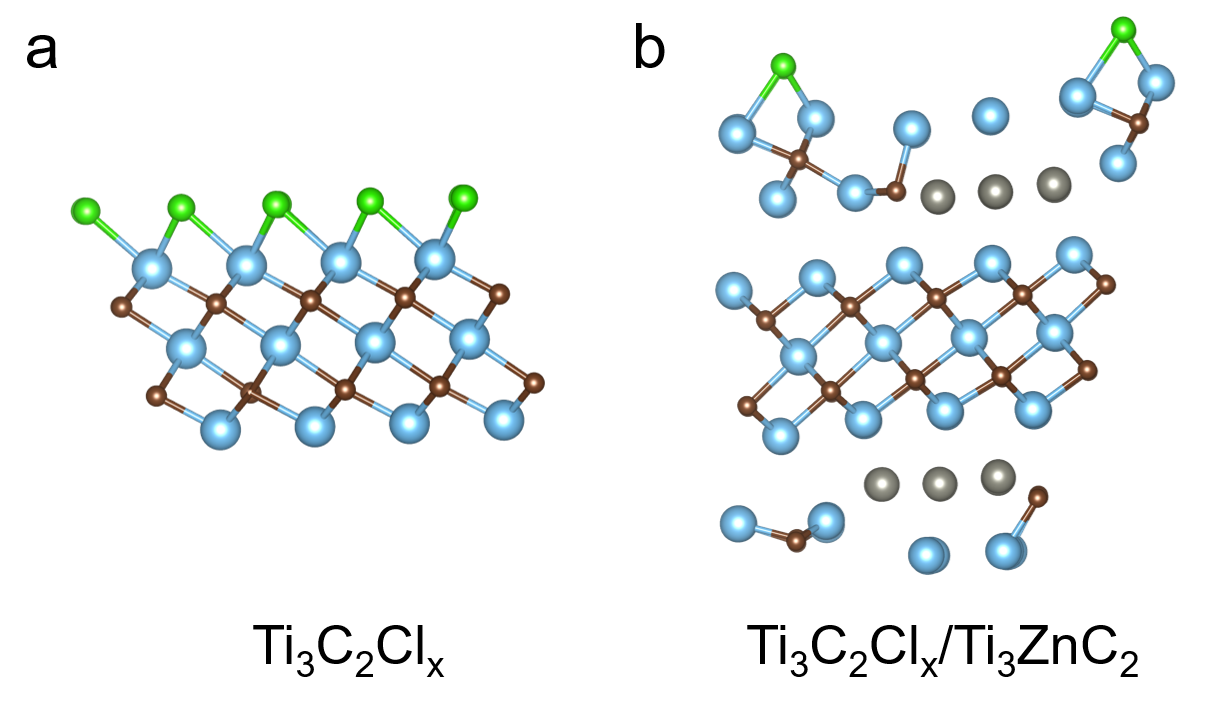


**Fig. S23** The optimized configuration for (a) Ti_3_C_2_Cl_x_ and (b) Ti_3_C_2_Cl_x_/Ti_3_ZnC_2_ heterostructure.


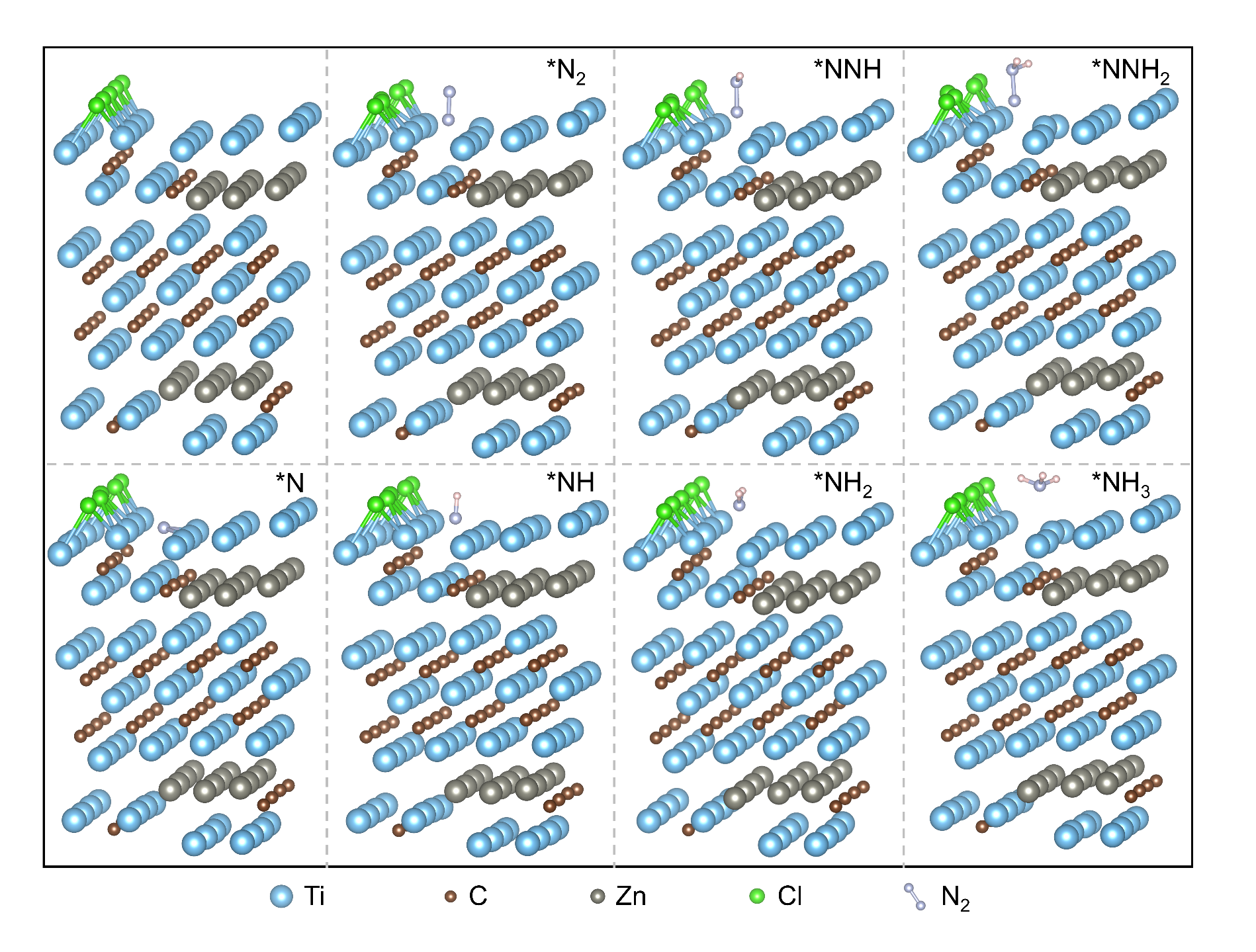


**Fig. S24** DFT-optimized adsorption geometries through distal pathways on Ti_3_C_2_Cl_x_/Ti_3_ZnC_2_ heterostructure.


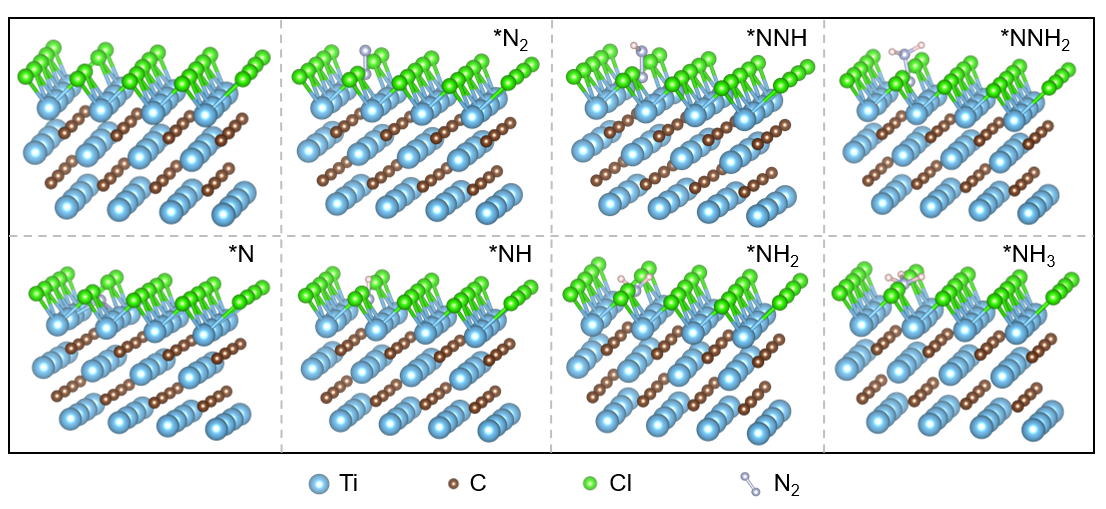


**Fig. S25** DFT-optimized adsorption geometries through distal pathways on Ti_3_C_2_Cl_x_.


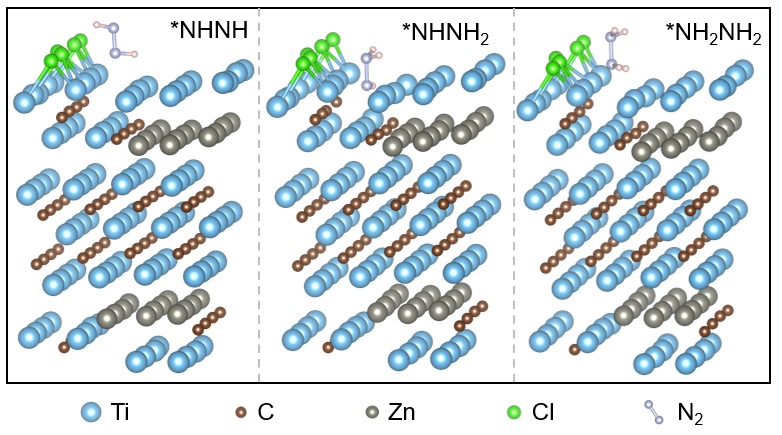


**Fig. S26** DFT-optimized adsorption geometries of *NHNH, *NHNH_2_, and *NH_2_NH_2_ on Ti_3_C_2_Cl_x_/Ti_3_ZnC_2_ heterostructure.


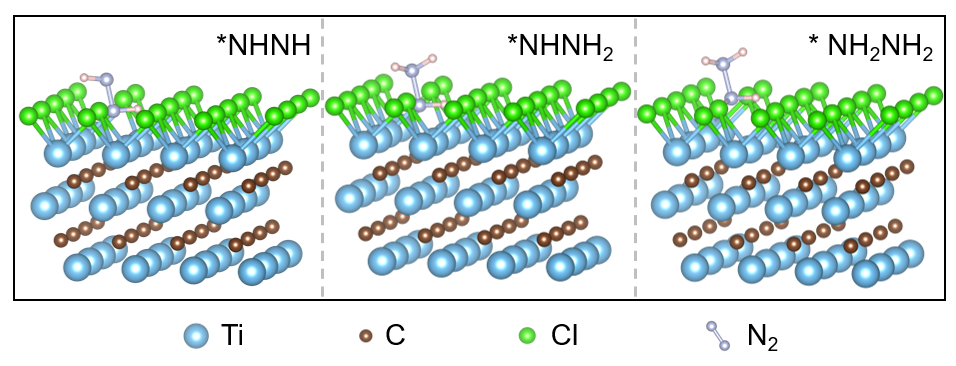


**Fig. S27** DFT-optimized adsorption geometries of *NHNH, *NHNH_2_, and *NH_2_NH_2_ on Ti_3_C_2_Cl_x_.


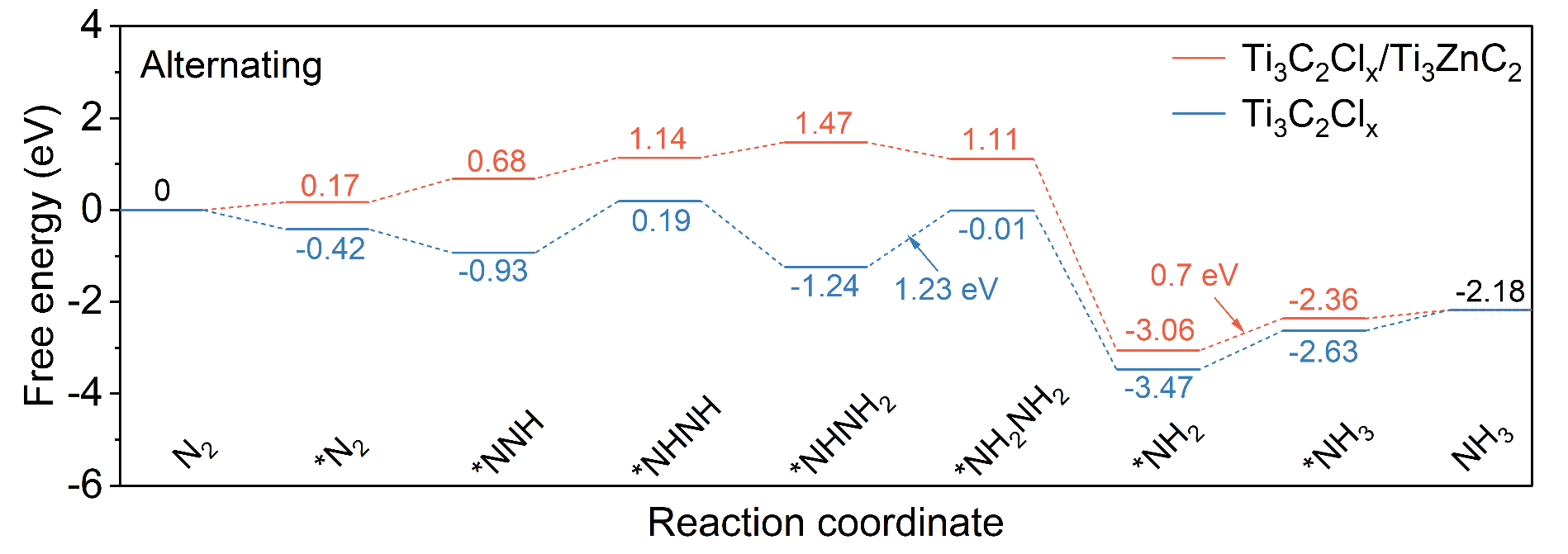


**Fig. S28** Calculated free energy diagram via alternating pathways on Ti_3_C_2_Cl_x_/Ti_3_ZnC_2_ heterostructure and Ti_3_C_2_Cl_x_.

**
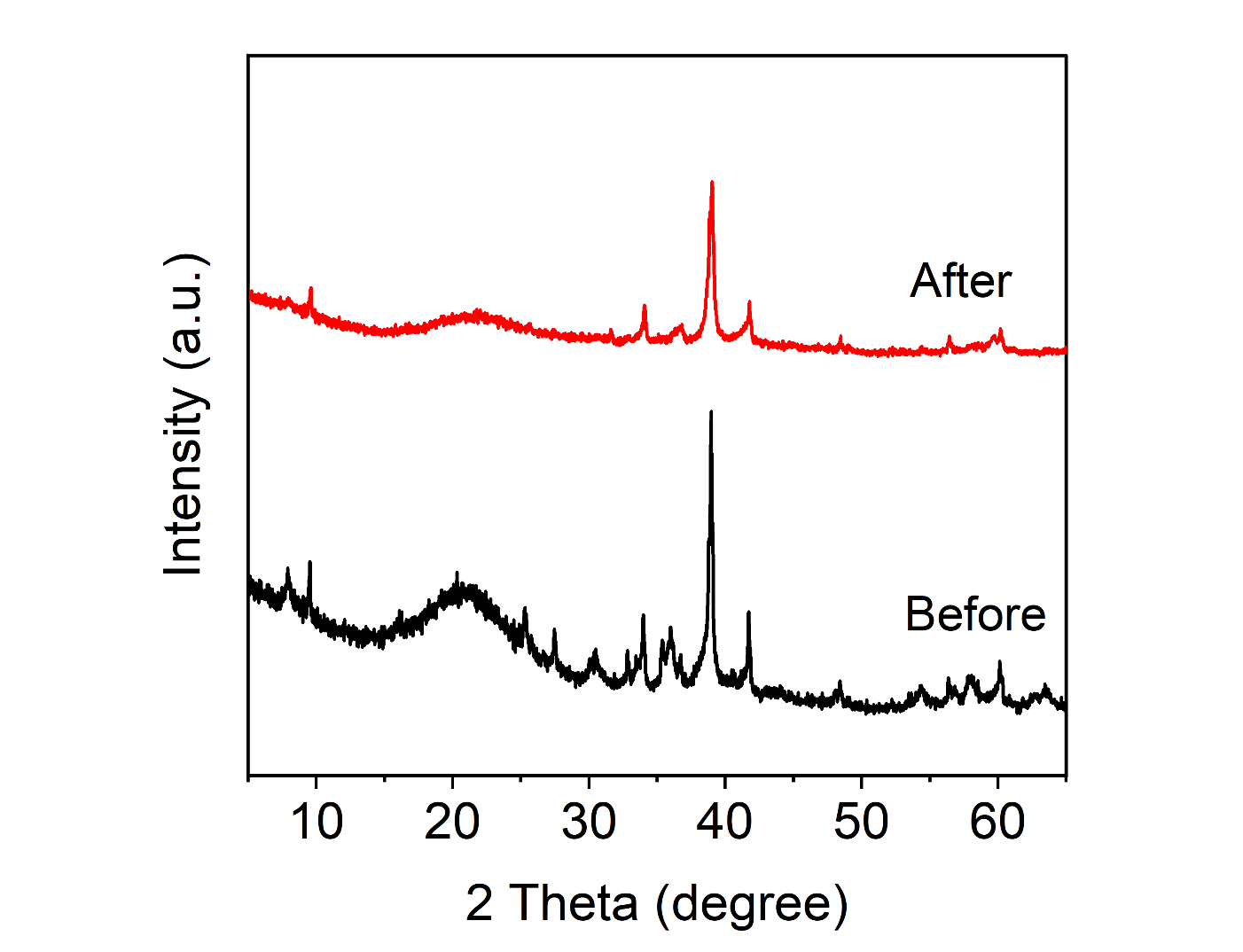
**

**Fig. S29** XRD patterns of Ti_3_C_2_Cl_x_/Ti_3_ZnC_2_ before and after continuous 5 h discharge test at 10 μA cm^−2^.

**Table S1** Comparison of electrocatalytic performance of T_3_C_2_Cl_x_/Ti_3_ZnC_2_ with recently reported NRR catalysts.

| Catalysts | Electrolyte | FE (%) | NH_3_ yield rate | Potential (vs. RHE) | Ref. |
| --- | --- | --- | --- | --- | --- |
| Ti_3_C_2_Cl_x_/Ti_3_ZnC_2_ | 0.1 M KOH | 38.1 | 20.1 µg h^−1^ mg^−1^ | −0.2 | This work |
| Sn@Ti_2_CT_X_/Ti_2_SnC-V | 0.1 M Na_2_SO_4_ | 15.57 | 28.4 µg h^−1^ mg^−1^ | −0.4 | [9] |
| Ti_3_C_2_ MXene/MAX | 0.1 M Na_2_SO_4_ | 36.9 | 2.73 µg h^−1^ cm^−2^ | −0.5 | [10] |
| MXene@NiCoB | 0.1 M Na_2_SO_4_ | 6.92 | 38.7 µg h^−1^ mg^−1^ | −0.4 | [11] |
| MoO_3-x_/MXene | 0.5 M LiClO_4_ | 22.3 | 95.8 µg h^−1^ mg^−1^ | −0.4 | [12] |
| 1T-MoS_2_@Ti_3_C_2_ | 0.1 M HCl | 10.94 | 30.33 µg h^−1^ mg^−1^ | −0.3 | [13] |
| MXene/Mn_3_O_4_ | 0.1 M Na_2_SO_4_ | 5.51 | 25.95 µg h^−1^ mg^−1^ | −0.5 | [14] |
| MXene/TiFeO_x_-700 | 0.05 M H_2_SO_4_ | 25.44 | 21.9 µg h^−1^ mg^−1^ | −0.2 | [15] |
| Ti_3_C_2_OH QDs | 0.1 M HCl | 13.30 | 62.94 µg h^−1^ mg^−1^ | −0.5 | [16] |
| Ti_3_C_2_T_x_ nanosheets | 0.5 M Li_2_SO_4_ | 5.78 | 4.7 µg h^−1^ cm^−2^ | −0.2 | [17] |
| BiOCl@Ti_3_C_2_T_x_ | 0.1 M HCl | 11.98 | 4.06 µg h^−1^ cm^−2^ | −0.1 | [18] |
| MnO_2_-Ti_3_C_2_T_x_ | 0.1 M HCl | 11.39 | 34.12 µg h^−1^ mg^−1^ | −0.55 | [19] |
| TiO_2_/Ti_3_C_2_T_x_ | 0.1 M HCl | 16.07 | 32.17 µg h^−1^ mg^−1^ | −0.55 | [20] |
| Zn^1^ N-C | 0.1 M KOH | 11.8 | 16.1 µg h^−1^ mg^−1^ | −0.3 | [21] |
| Zn nanosheets | 0.5 M LiClO_4_ | 11.7 | 25.3 µg h^−1^ mg^−1^ | −0.5 | [22] |
| W_2_N_3_ | 0.1 M KOH | 11.67 | 11.66 µg h^−1^ mg^−1^ | −0.2 | [23] |
| SA Ru-Mo_2_CT_x_ | 0.5 M K_2_SO_4_ | 25.77 | 40.57 µg h^−1^ mg^−1^ | −0.3 | [24] |

**Table S2** Comparison of power density and NH_3_ yield of T_3_C_2_Cl_x_/Ti_3_ZnC_2_-based Zn-N_2_ battery with other recently reported Zn-N_2_ batteries.

| Catalysts | Electrolyte | Power density | NH_3_ yield | Ref. |
| --- | --- | --- | --- | --- |
| Ti_3_C_2_Cl_x_/Ti_3_ZnC_2_ | 1 M KOH | 36.5 μW cm^−2^ | 13.1 µg h^−1^ mg^−1^ | This work |
| Ti_3_C_2_Cl_x_ | 6 M KOH | 13.98 μW cm^−2^ | 9.17 µg h^−1^ mg^−1^ | [25] |
| nano-Cu | 0.1 M KOH | 10.1 μW cm^−2^ | 0.125 µg h^−1^ cm^−2^ | [26] |
| VN@NSC-900 | 0.1 M KOH | 16.42 μW cm^−2^ | 0.172 µg h^−1^ cm^−2^ | [27] |
| NbS_2_ nanosheets | 0.1 M HCl | 0.31 mW cm^−2^ | N.A. | [28] |
| Fe_1.0_HTNs | 0.1 M KOH | 27.65 μW cm^−2^ | 0.137 µg h^−1^ cm^−2^ | [29] |
| CoPi/HSNPC | 1 M KOH | 0.33 mW cm^−2^ | 11.58 µg h^−1^ mg^−1^ | [30] |
| CoPi/NPCS | 1 M KOH | 0.49 mW cm^−2^ | 14.7 µg h^−1^ mg^−1^ | [31] |
| Vs-FePS_3_ NSs | N.A. | 2.6 mW cm^−2^ | N.A. | [32] |
| OV-Ti_2_O_3_ | 0.1 M HCl | 1.02 mW cm^−2^ | 4.3 µg h^−1^ mg^−1^ | [33] |

**References**

[1] M. Li, J. Lu, K. Luo, Y. Li, K. Chang, K. Chen, J. Zhou, J. Rosen, L. Hultman, P. Eklund, P.O.A. Persson, S. Du, Z. Chai, Z. Huang, Q. Huang, *J. Am. Chem. Soc.* **2019**, *141*, 4730.

[2] D. Zhu, L. Zhang, R.E. Ruther, R.J. Hamers, *Nat. Mater.* **2013**, *12*, 836.

[3] B. Yu, H. Li, J. White, S. Donne, J.B. Yi, S.B. Xi, Y. Fu, G. Henkelman, H. Yu, Z.L. Chen, T.Y. Ma, *Adv. Funct. Mater.* **2020**, *30*, 1905665.

[4] G.W. Watt, J.D. Chrisp, *Anal. Chem.* **1952**, *24*, 2006.

[5] G. Kresse, J. Hafner, *Phys. Rev. B* **1993**, *48*, 13115.

[6] G. Kresse, J. Hafner, *Phys. Rev. B* **1993**, *47*, 558.

[7] J.P. Perdew, K. Burke, M. Ernzerhof, *Phys. Rev. Lett.* **1996**, *77*, 3865.

[8] G. Kresse, D. Joubert, *Phys. Rev. B* **1999**, *59*, 1758.

[9] X.Y. Dai, Z.Y. Du, Y. Sun, P. Chen, X.G. Duan, J.J. Zhang, H. Li, Y. Fu, B.H. Jia, L. Zhang, W.H. Fang, J.S. Qiu, T.Y. Ma, *Nano Micro Lett.* **2024**, *16*, 89.

[10] K. Ba, D.D. Pu, X.Y. Yang, T. Ye, J.H. Chen, X.R. Wang, T.S. Xiao, T. Duan, Y.Y. Sun, B.H. Ge, P. Zhang, Z.Q. Liang, Z.Z. Sun, *Appl. Catal. B Environ.* **2022**, *317*, 121755.

[11] C. Wang, Q.C. Wang, K.X. Wang, M. De Ras, K. Chu, L.-L. Gu, F. Lai, S.-Y. Qiu, H. Guo, P.-J. Zuo, J. Hofkens, X.-D. Zhu, *J. Energy Chem.* **2023**, *77*, 469.

[12] K. Chu, Y.J. Luo, P. Shen, X.C.A. Li, Q.Q. Li, Y.L. Guo, *Adv. Energy Mater.* **2022**, *12*, 2103022.

[13] X. Xu, B. Sun, Z. Liang, H. Cui, J. Tian, *ACS Appl. Mater. Interfaces* **2020**, *12*, 26060.

[14] C. Wang, X.D. Zhu, P.J. Zuo, *Chem. Eng. J.* **2020**, *396*, 125163.

[15] Y. Guo, T. Wang, Q. Yang, X. Li, H. Li, Y. Wang, T. Jiao, Z. Huang, B. Dong, W. Zhang, J. Fan, C. Zhi, *ACS Nano* **2020**, *14*, 9089.

[16] Z. Jin, C. Liu, Z. Liu, J. Han, Y. Fang, Y. Han, Y. Niu, Y. Wu, C. Sun, Y. Xu, Adv. Energy Mater. **2020**, *10*, 2000797.

[17] Y. Luo, G.F. Chen, L. Ding, X. Chen, L.-X. Ding, H. Wang, Joule **2019**, *3*, 279.

[18] Y. Wang, M. Batmunkh, H. Mao, H. Li, B. Jia, S. Wu, D. Liu, X. Song, Y. Sun, T. Ma, *Chin. Chem. Lett.* **2022**, *33*, 394.

[19] W. Kong, F. Gong, Q. Zhou, G. Yu, L. Ji, X. Sun, A.M. Asiri, T. Wang, Y. Luo, Y. Xu, *J. Mater. Chem. A* **2019**, *7*, 18823.

[20] Y. Fang, Z. Liu, J. Han, Z. Jin, Y. Han, F. Wang, Y. Niu, Y. Wu, Y. Xu, *Adv. Energy Mater.* **2019**, *9*, 1803406.

[21] Y. Kong, Y. Li, X. Sang, B. Yang, Z. Li, S. Zheng, Q. Zhang, S. Yao, X. Yang, L. Lei, S. Zhou, G. Wu, Y. Hou, *Adv. Mater.* **2022**, *34*, 2103548.

[22] Q. Li, J. Wang, Y. Cheng, K. Chu, *J. Energy Chem.* **2021**, *54*, 318.

[23] H. Jin, L. Li, X. Liu, C. Tang, W. Xu, S. Chen, L. Song, Y. Zheng, S.Z. Qiao, *Adv. Mater.* **2019**, *31*, e1902709.

[24] W. Peng, M. Luo, X. Xu, K. Jiang, M. Peng, D. Chen, T.S. Chan, Y. Tan, *Adv. Energy Mater.* **2020**, *10*, 2001364.

[25] Y. Wang, Y. Sun, T. Ma, P. Chen, Y. Fu, H. Li, B. Jia, X. Duan, W. Zhang, W. Fang, J. Yan, Q. Yang, L. X. Ding, D. Su, J. Qiu, *Adv. Funct. Mater.* **2025**, e20841.

[26] C. Du, Y. Gao, J. Wang, W. Chen, *Chem. Commun.* **2019**, *55*, 12801.

[27] X. W. Lv, Y. Liu, Y. S. Wang, X. L. Liu, Z. Y. Yuan, *Appl. Catal. B Environ.* **2021**, *280*, 119434.

[28] H. Wang, J.C. Si, T. Y. Zhang, Y. Li, B. Yang, Z. J. Li, J. Chen, Z. H. Wen, C. Yuan, L. C. Lei, Y. Hou, *Appl. Catal. B Environ.* **2020**, *270*, 118892.

[29] X. W. Lv, X. L. Liu, L. J. Gao, Y. P. Liu, Z. Y. Yuan, *J. Mater. Chem. A* **2021**, *9*, 4026.

[30] J. T. Ren, L. Chen, Y. P. Liu, Z. Y. Yuan, *J. Mater. Chem. A* **2021**, *9*, 11370.

[31] J. T. Ren, L. Chen, H. Y. Wang, Z. Y. Yuan, *ACS Appl. Mater. Interfaces* **2021**, *13*, 12106.

[32] H. Wang, Z. Li, Y. Li, B. Yang, J. Chen, L. Lei, S. Wang, Y. Hou, *Nano Energy* **2021**, *81*, 105613.

[33] H. J. Chen, Z. Q. Xu, S. J. Sun, Y. S. Luo, Q. Liu, M. S. Hamdy, Z. S. Feng, X. P. Sun, Y. Wang, *Inorg. Chem. Front.* **2022**, *9*, 4608.
